# Physiological elasticity and topography modulate *in vitro* prostate cancer cell behavior

**DOI:** 10.1016/j.isci.2026.117623

**Published:** 2026-09-30

**Authors:** Marco Hoffmann, Richard Paul, Anna Herrmann, Sven Gerlach, Paul Bacmeister, Thomas Frank Ermler, Marie Book, Radu Alexa, Axel Honné, Julian Peter Müller, Julia Kristin Brach, Petra Fischer, Jennifer Kranz, Matthias Wessling, Matthias Saar

**Affiliations:** 1 Department of Urology and Pediatric Urology, University Hospital RWTH Aachen, Aachen, Germany; 2 Center for Integrated Oncology (CIO), University Hospital RWTH Aachen, Aachen, Germany; 3 Chemical Process Engineering AVT.CVT, RWTH Aachen University, Aachen, Germany; 4 Scientific Workshop, University Hospital RWTH Aachen, Aachen, Germany; 5 Institute of Clinical Pharmacology, RWTH Aachen University, Aachen, Germany; 6 Department of Otorhinolaryngology-Head and Neck Surgery, University Hospital RWTH Aachen, Aachen, Germany

**Keywords:** durotaxis, topotaxis, hydrogel, *in vitro*, translational, chemosensitivity, prostate, cancer, modulation

## Abstract

Tissue elasticity and microenvironment are a hallmark of cancer, particularly in prostate cancer. Here, we investigate, how physiologically relevant mechanical properties affect prostate cancer cells using 2D-poly(dimethylsiloxane) (PDMS) and topographic 2.5D-poly(ethylene glycol) diacrylate-glycidyl methacrylate hydrogel surfaces. Substrates reproducing the stiffness of tumor cell niches, altered cell morphology, metabolic activity, migration, cytokine expression, and therapeutic response. Elasticity-dependent effects included up to a 1.5-fold increase in LDH activity with concurrent cell proliferation, reduced migration rates with increased paxillin expression, and enhanced chemokine-mediated invasion. Adaptation to physiological elasticities was also associated with up to 90 % reduction in responsiveness to docetaxel and enzalutamide. Importantly, elasticity-adapted substrates supported primary prostate cancer cell growth while preserving characteristic expression profiles. These findings demonstrated that mechanical microenvironmental cues substantially shape prostate cancer cell phenotypes and highlight their importance for developing more physiologically relevant and translationally informative *in vitro* models.

## Highlights

• Multi-elasticity models reproduce mechanical features of prostate tumor niches
• Physio-elastic substrates alter cell migration, invasion, and signaling
• Elastic microenvironments reduce sensitivity to docetaxel and enzalutamide
• Primary cultures retain patient-associated AR and PSMA expression patterns

## Graphical abstract

**Figure:**
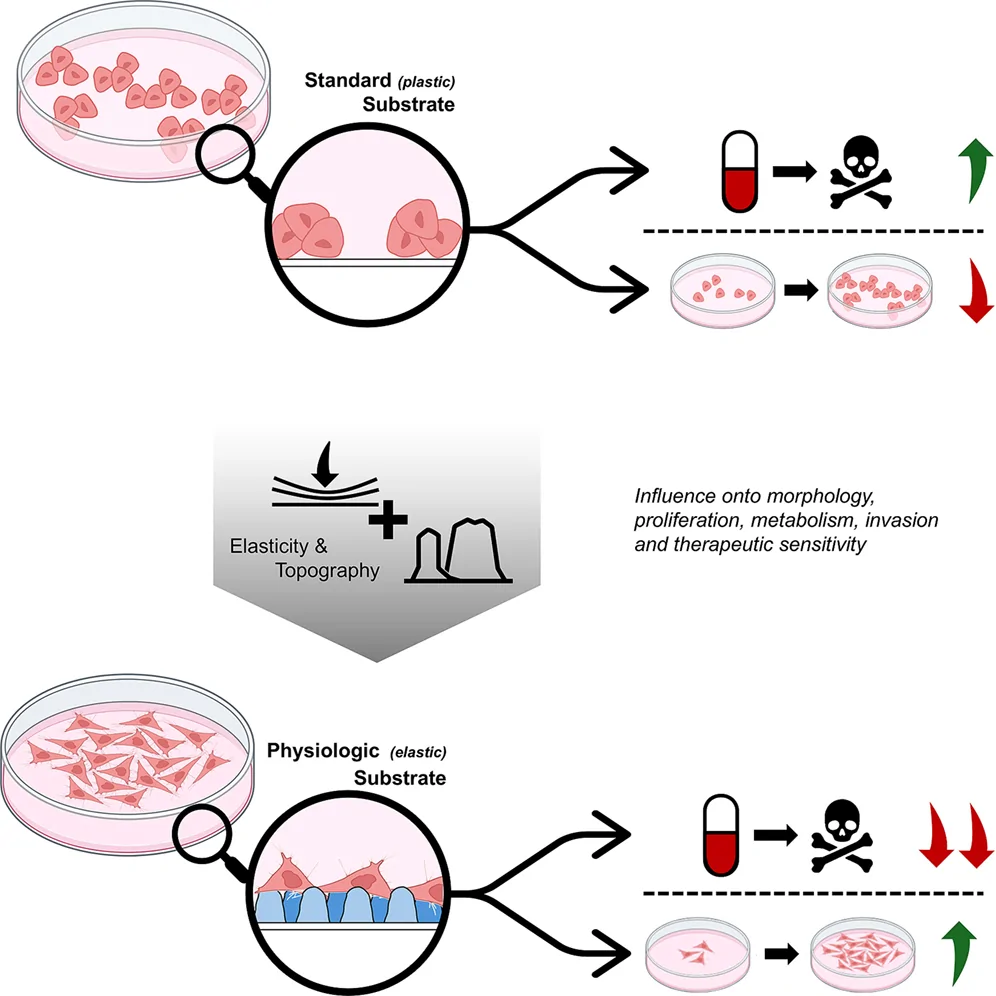

## Teaser

Cancer systems biology; Cell biology

## Introduction

Translational cancer research permanently faces the challenge of striking a balance between the complex pathobiology of human tumors and the necessary simplicity as well as reproducibility of laboratory methodologies. Tumors display extensive heterogeneity, from an intricate composition of cancer cells, resident stromal and immune cell populations, the extracellular matrix (ECM) and the variability in nutrient and oxygen supply.^1^^,^^2^^,^^3^ Primary cultures of such tumors pose unique challenges, and in some cases cannot be implemented for *in vitro* studies, with prostate cancer providing a particularly relevant example to explore these limitations.^4^^,^^5^^,^^6^

Prostate cancer ranks as the second most common cancer globally and the fifth leading cause of cancer-related deaths among men.^7^ In 2020, approximately 1.4 million new cases of prostate cancer were diagnosed, representing 14 % of all male cancer cases, and around 375,000 men died from this disease. By 2040, demographic shifts are projected to nearly double the number of prostate cancer cases and deaths worldwide.^8^

The formation of prostate cancer is accompanied by tissue hardening, allowing clinically significant prostate cancer to be identified by palpation or transrectal ultrasound (TRUS) examination and TRUS elastography.^9^^,^^10^^,^^11^ In research, *in vitro* models are used as an initial step for the fundamental characterization of diseases and for the development of innovative diagnostic and therapeutic approaches. However, traditional *in vitro* cell culture methods often utilize rigid, two-dimensional substrates, which fail to accurately mimic the dynamic and complex environment of human tissues and its disease-related alterations.^12^^,^^13^ Physiological cell culture substrates are essential to provide a more realistic representation of the *in vivo* conditions for cancer cells,^14^^,^^15^ cancer-associated fibroblasts,^16^ and healthy epithelial cells.^17^ These substrates provide essential mechanotransduction cues, enabling a more physiologically and pathologically relevant representation of the *in vivo* mechanical and microenvironmental landscape, thereby improving the translational value of *in vitro* studies.^13^^,^^18^

The mechanical properties of organs and their surrounding tissues are critical for cellular functionality, differentiation, and interaction.^19^^,^^20^ Tissue elasticity varies widely, ranging from approximately 1 to over 10,000 kPa. In the context of prostate cancers, tumor microenvironment (TME), specifically in its mechanical TME (mTME) and its most common metastatic tumor niches (MTNs), distinct differences in mechanical properties can be observed. The elasticity of the prostate itself increases under pathological progression, from values of approximately 50 kPa in healthy tissue to around 90 kPa in malignant states.^10^ A similar trend is evident in lymph nodes, where physiological stiffness (∼40 kPa) contrasts with the elevated values found in pathological tissues (∼60 kPa).^21^ Bone marrow, another frequent MTN of prostate cancer, exhibits significantly lower elasticity, ranging from 0.5 to 1.5 kPa, whereas trabecular bone displays markedly higher stiffness values between 2,000 and 10,000 kPa.^22^^,^^23^ The response to mechanical stiffness of the mTME and relevant MTN, a phenomenon known as durotaxis, can be replicated *in vitro* using 2D-poly(dimethylsiloxane) (PDMS) substrates, reaching elastic moduli of up to 1,600 kPa.^24^ In contrast, the structural guidance of tissue architecture (2.5D), referred to as topotaxis, can be mimicked using photolithographically crosslinked materials such as poly(ethylene glycol) diacrylate-glycidyl methacrylate (PEGDA-GMA), achieving stiffness levels of up to 350 kPa.^25^ Translational research addresses the need for accurate preclinical models that reflect the complexity of prostate cancer. Current models, including prostate cancer-derived cell lines from different metastatic sites (e.g., PC3, bone; LNCaP, lymph; VCaP, bone marrow), offer cost-effective drug screening but only limited representation of primary prostate cancer phenotypes.^26^ In contrast, three-dimensional (3D) cultures better mimic tumor architecture and metabolism, thus enhancing the physiological relevance of drug-testing by recapitulating characteristics of the TME.^27^^,^^28^^,^^29^^,^^30^ The patient-derived xenotransplant cell line LuCaP and organoids strive to replicate tumor heterogeneity for personalized medicine, yet challenges in reproducibility and fidelity to original tissues persist.^31^^,^^32^^,^^33^

To address this gap in the interplay of topo- and durotactic cues in preclinical *in vitro* cell cultures, we employed 2D-PDMS and topographic 2.5D-PEGDA-GMA hydrogel surfaces with physiological elasticity to investigate functional characteristics of prostate cancer cell lines. We reconstructed key features of the mTME on cell-culture substrates, developed a multi-elasticity-migration-assay, adapted Boyden Chamber assays to reproduce mTME conditions more physiologically and conducted experiments with the immortalized prostate cancer-derived cell lines PC3, LNCaP, and VCaP. The effects of physiologically adapted substrates, defined by their topographic and elastic properties, were analyzed on cell line proliferation, migration, metabolic activity, morphological features, cytoskeletal organization, intercellular communication, and chemosensitivity. In addition, the influence of physiologically inspired culture systems on the resulting cultivation of primary prostate tumor cells was characterized in comparison to standard culture substrates.

We hypothesize that the replication of durotactic and topotactic cues in preclinical cell culture models might be a necessity to achieve physiological relevant outcomes. We envision that advancing preclinical methods, such as *in vitro* assays capable of replicating physiological mTME features, could enhance translational research and improve individual patient-derived tumor cultures.

## Results

### Cell proliferation, metabolism, and morphology parameters increase with substrate elasticity

We characterized three prostate cancer cell lines, LNCaP, PC3, and VCaP, cultured on elastomer substrates (2D-PDMS) with mechanical tumor microenvironment (mTME)-specific elasticities.

Cell quantification indicated a significant improvement in adherent cell growth on prostate cancer mTME substrates (90 kPa) for LNCaP (2.0-fold) and PC3 (2.7-fold) compared with cells cultivated on plastic substrates. VCaP cells showed a similar, yet non-significant trend with 1.4-fold higher cell numbers (see Figure 1A).

**Figure 1:**
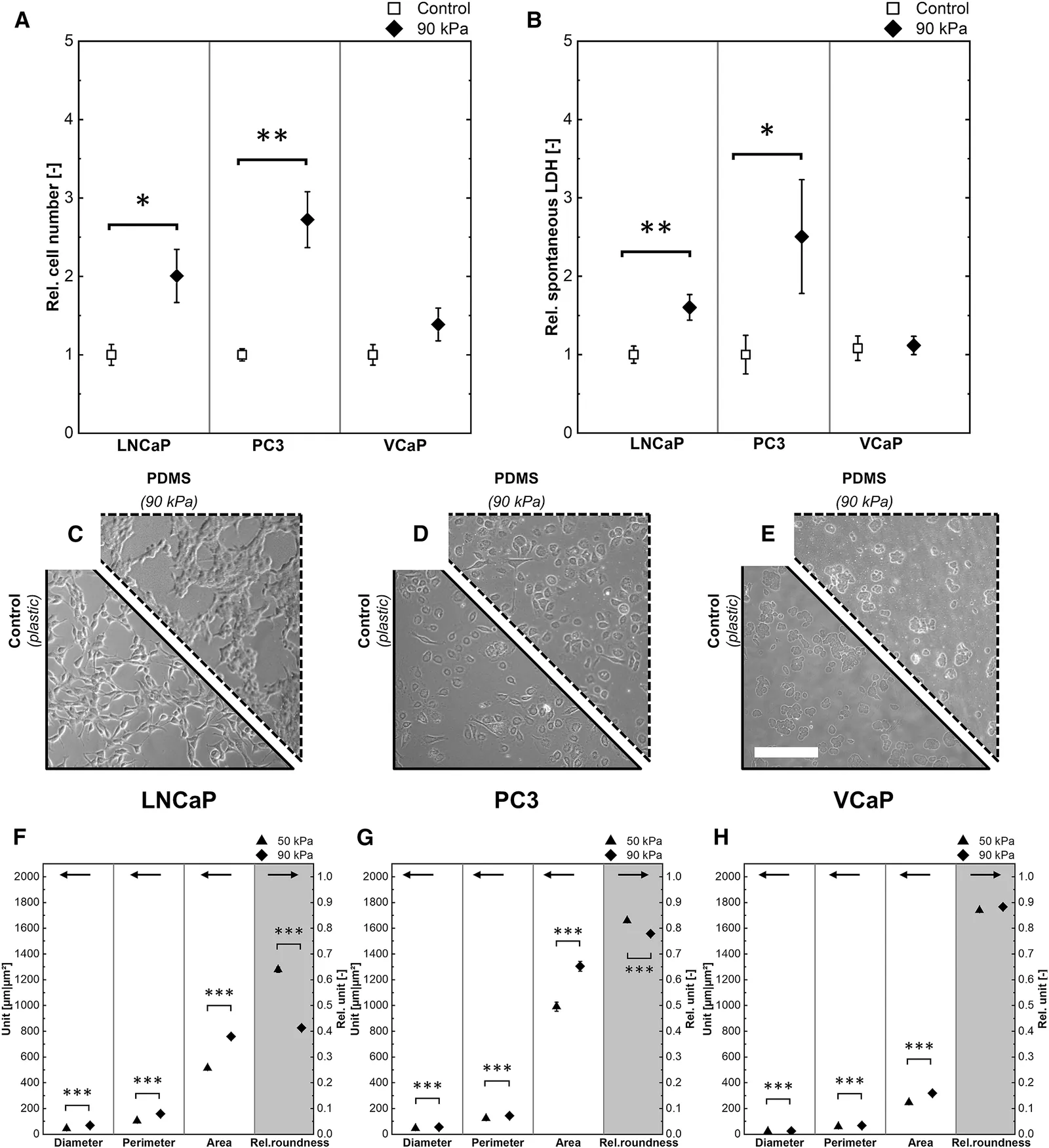
Cultivation of prostate cancer cell lines on plastic and physio-elastic substrates (A and B) The CASY-cell counter was used to quantify cell numbers on plastic (Control, ⧠) and physio-elastic substrates (PDMS) (90 kPa, ◆) after 48 h of cultivation (A) and LDH activity to characterize the metabolic activity of prostate cancer cell lines (B). Cultivation of LNCaP, PC3, and VCaP under standard culture conditions (Control, ⧠) and 90 kPa, ◆ induced qualitative changes in cell morphology. (C–H) Cultivation of LNCaP, PC3, and VCaP was performed on 50 and 90 kPa, ◆. Cell morphological parameters were analyzed by diameter, perimeter, area (F–H, left *y* axis) and level of roundness (F–H, right *y* axis). A roundness value of 1.0 represents an ideal circular shape. Data are represented as mean ± SEM. Asterisks indicate significance levels determined by Mann-Whitney U test (MWU): ∗*p* ≤ 0.05, ∗∗*p* ≤ 0.01, and ∗∗∗*p* ≤ 0.001, based on at least three independent experiments. A minimum of 150 cells were analyzed (F–H). Scale bars, 150 μm.

Analysis of LDH activity revealed a significant 1.6-fold increase with a narrowing of variation for LNCaP cells on elastomer substrates compared with plastic substrates. For PC3, an even more pronounced 2.5-fold increase in LDH activity was observed. VCaP showed comparable results toward elevated LDH activities on prostate cancer mTME substrates (see Figure 1B).

Microscopic analysis of cell cultures after 48 h already indicated morphological alterations between cells cultivated on plastic and 90 kPa PDMS substrates (Figures 1C–1E). More detailed quantification of morphological parameters (see Figures 1F–1H) was performed on healthy prostate (50 kPa) and prostate cancer-specific mTME substrates (90 kPa) and compared with cultivation on plastic controls. The analysis of diameter, perimeter, area, and roundness revealed a significant increase over all analyzed parameters for almost the entirety of the prostate cancer cell types when cultured on the prostate cancer-specific elasticities. LNCaP exhibited the most pronounced increase in morphological parameters (diameter, 1.56-fold; perimeter, 1.52-fold; and area, 1.48-fold). Additionally, PC3 (diameter, 1.21-fold; perimeter, 1.17-fold; area, 1.32-fold) and VCaP (diameter, 1.13-fold; perimeter, 1.15-fold; area, 1.32-fold) showed significant changes on physiological substrate, but the increases were more moderate than those observed for LNCaP. Notably, cell roundness was significantly reduced for LNCaP and PC3 (see Figures 1F and 1G), while VCaP maintained a consistent, non-significant level when cultured on prostate cancer mTME substrates.

These alterations could also be confirmed in representative analyses of tissue specimens. Given the well-characterized alterations of elasticity and topography in the mTME of prostate cancer progression, we investigated the impact of these environmental changes on cell morphology and the actin cytoskeleton in surgical tissue samples. For this purpose, six tissue samples from two patients each with histologically confirmed prostate cancer states (benign; low-risk = Gleason score 7; high-risk = Gleason score 9) were included (see Figures S1A–S1C). The quantitative analysis of morphological parameters (see Figure S1D), actin intensity (see Figure S1E) and alignment (see Figure S1F) revealed distinct changes across benign and malignant states. Although cellular roundness remained relatively consistent across analyzed specimens, a significant decline in cell area was observed with an increasing Gleason score. The mean actin signal intensity did not exhibit a linear trend with increasing Gleason Score. However, the variation in signal intensity decreased significantly, indicated by a narrowing of the deviation.

### Cellular cytoskeleton adapts to substrate elasticity depending on the cell’s origin

To examine the effect of substrate elasticity on cellular cytoskeletal organization, prostate cancer cell lines were cultivated on physiological elasticities specific to their associated MTNs and the local prostate cancer mTME. LNCaP were cultured on elasticities associated with lymph node MTN-elasticity (50 kPa) and are compared against the local prostate cancer mTME (90 kPa). PC3 and VCaP were analyzed on elasticities associated with bone marrow (1 kPa), trabecular bone (1,600 kPa) and the local prostate cancer mTME (90 kPa).

The characterization of the cytoskeletal components, actin and paxillin, is shown in Figures 2A–2C as immunofluorescence staining. High actin expression can be interpreted as indicative of a more dynamic, remodeling cell phenotype, whereas elevated paxillin expression is commonly associated with more sedentary, adhesion-stabilized cells. To substantiate these phenotypic indications, we assessed cell migration and invasion in detail.

**Figure 2:**
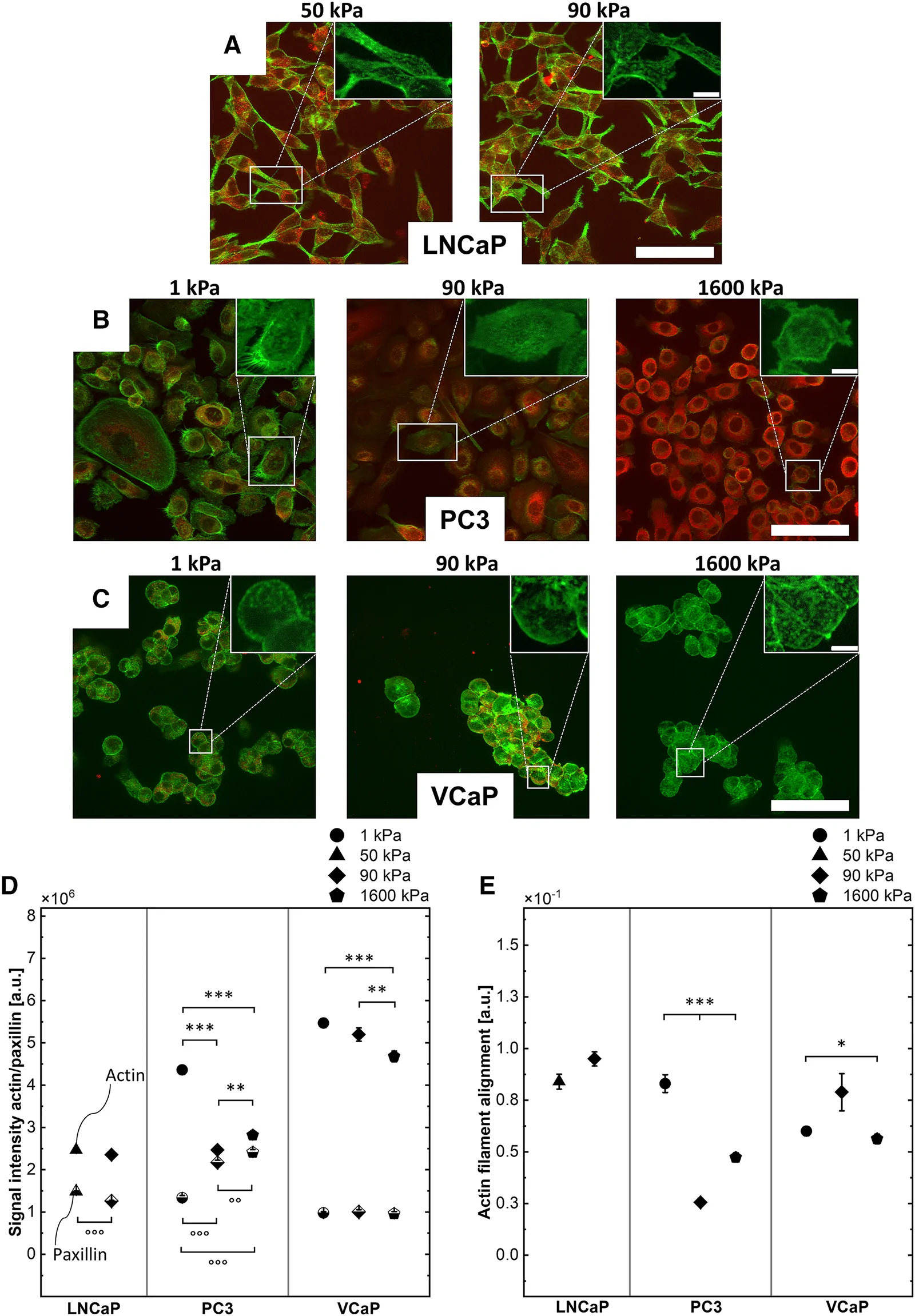
Cytoskeleton of prostate cancer cell lines on physio-elastic substrates (A–E) Cultivation of prostate cancer cell lines was conducted on physiological elasticities specific to their MTN (A–C). Immunofluorescence staining images in (A–C) show cytoskeletal protein expression of actin (green) and paxillin (red). The signal intensities of the expressed cytoskeletal proteins actin and paxillin of each respective cell line are shown in (D). The intensity of actin is represented by fully filled symbols, while paxillin is indicated by semi-filled symbols, corresponding to each substrate stiffness (1 kPa ●/◒, 50 kPa ▲/, 90 kPa ◆/⬙, 1600 kPa ⬟/). The actin filament alignment is shown in (E). Data are represented as mean ± SEM. Asterisks and circles indicate significances for actin expression, circles indicate significances of paxillin levels determined by Mann-Whitney U test (MWU): *p* ≤ 0.05 (∗/○), *p* ≤ 0.01 (∗∗/○○), and *p* ≤ 0.001 (∗∗∗/○○○), based on at least 150 different cell analyses. Scale bars, 100 μm; insert scale bars, 10 μm.

The actin intensity as well as its alignment did not exhibit significant differences when cultured on both elasticities. LNCaP showed a significant 1.17-fold higher paxillin intensity when cultured on 50 kPa compared with 90 kPa substrates (see Figures 2D and 2E). PC3 exhibited significant changes in actin and paxillin intensity as well as actin filament alignment between 1, 90, and 1,600 kPa conditions. Here, we observed a distinct and significant shift in expression patterns, transitioning from high actin expression on non-physiological soft, to high paxillin expression on physiological stiff substrates (see Figure 2A). According to this, the actin alignment of the PC3 cells showed significant changes for all substrate elasticities (see Figure 2B). Comparable with PC3, VCaP showed a significant decrease in actin expression when being cultured on its native elasticities but did not show increased paxillin expression with higher elastomer substrate stiffness (1, 90, and 1,600 kPa). The actin filament alignment of VCaP was significantly reduced on 1,600 kPa substrates compared with 1 kPa, whereas a similar alignment trend was observed on 90 kPa (see Figure 2C).

### Modulation of cell migration, invasion, communication, and chemosensitivity by substrate elasticity

#### Physiologic MTN substrates diminish cell migration

Cell migration and invasion are key parameters for studying mechanisms associated with metastatic progression *in vitro*. To characterize the influence of physiologically relevant substrate mechanics on these processes, the migratory behavior of LNCaP prostate cancer cells and non-malignant PNT2 prostate epithelial cells was first assessed on plastic and physio-elastic substrates in a co-culture elasticity migration assays (CEMAs), to evaluate whether substrate mechanics and cell-cell interactions modulate migration.

LNCaP cells cultured on plastic controls exhibited consistently higher migration velocities than cells cultured on 50 or 90 kPa. Interestingly, co-culture with PNT2 cells reversed this effect, resulting in significantly increased migration rates of LNCaP cells on physio-elastic substrates (see Figure S2). These findings demonstrate that physiologically relevant substrate mechanics not only directly regulate tumor cell migration but also influence the cellular interactions that govern migratory behavior. Given the pronounced effects of substrate elasticity and co-culture conditions, these observations were further investigated using a multi-elasticity migration assay (MEMA) incorporating physiologically relevant stiffness gradients of the MTN (see Figures 3A–3C) and a physiologically relevant Boyden Chamber invasion model.

**Figure 3:**
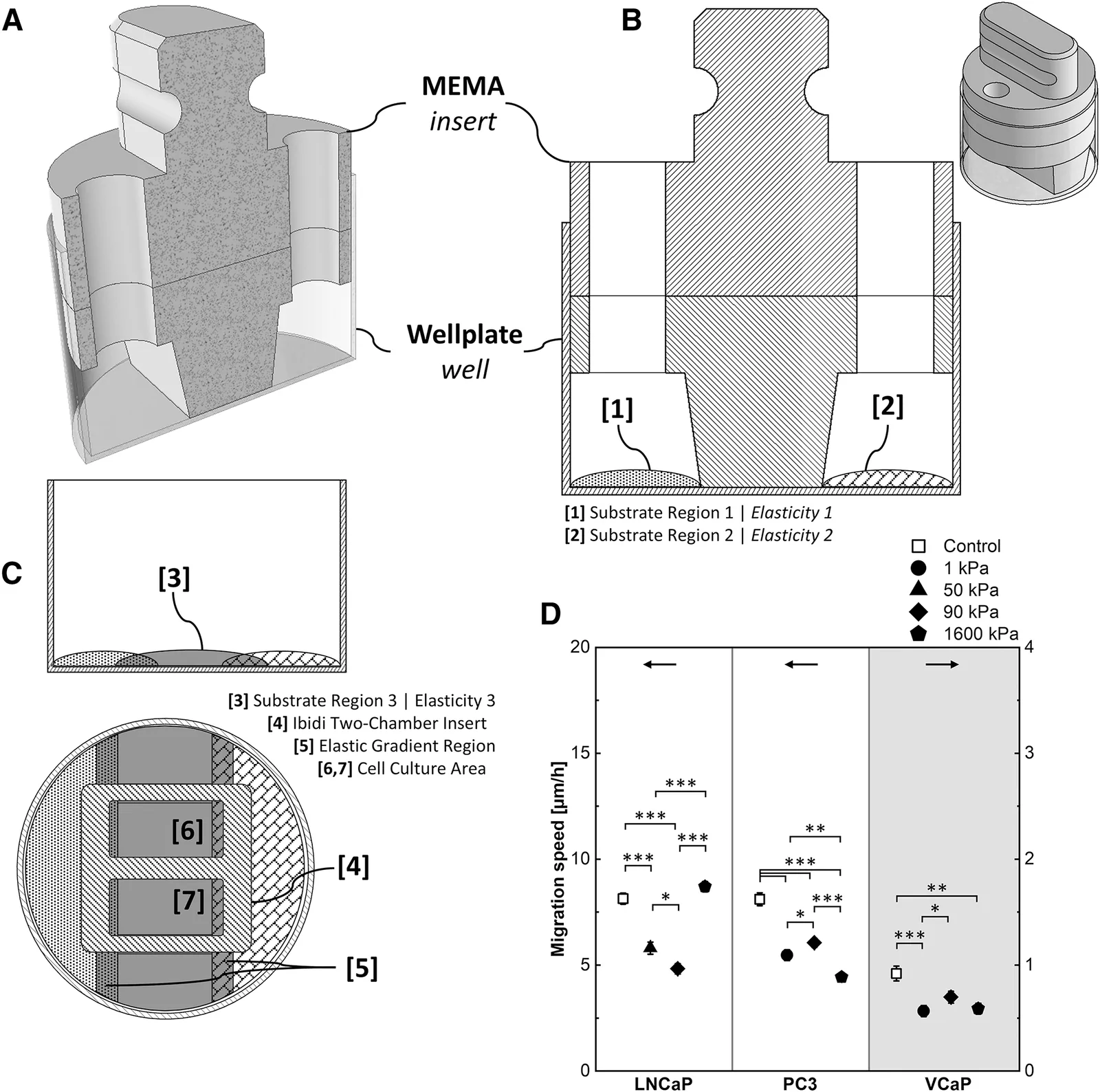
Characterization of migration activity on different substrate elasticities using a self-established multi-elasticity migration assay (MEMA) (A–C) The MEMA insert fits a standard 12-well plate (A). After both chambers are filled with the desired substrate elasticity (SE) (SE1, [1]; SE2, [2]) (B), the MEMA stamp is removed, and the third substrate composition is pipetted in between the other two (SE3, [3]) to form a diffusive gradient of elasticity (SE5, [5]) (C). For the LNCaP cell line, lymph node MTN (50 kPa, SE1, ▲) and prostate cancer mTME substrates (90 kPa, SE2, ◆) with 1,600 kPa in the starting substrate region (SE3, ⬟) are used. For PC3 and VCaP cell lines, bone marrow MTN (1 kPa, SE1, ●), prostate cancer mTME (90 kPa, SE2, ◆) and 1,600 kPa (SE3, ⬟) were used. Cells were seeded in the culture area ([6, 7]), and the insert ([4]) was removed after 48 h of cultivation (C). All cell lines were additionally cultured on plastic controls (Control, ⧠) and characterized for migrative activity. (D) Data are represented as mean ± SEM. Asterisks indicate significance levels determined by Mann-Whitney U test (MWU): ∗*p* ≤ 0.05, ∗∗*p* ≤ 0.01, and ∗∗∗*p* ≤ 0.001, based on at least three independent experiments. Migration speed was quantified at 10 positions in at least 20 images for each culture condition.

The MEMA’s substrate regions are matched with the cell line’s individual MTN and prostate cancer mTME. The central substrate elasticity (SE3) was set to 1,600 kPa to approximate the rigidity of plastic and thereby initiate cell migration from a surface closely resembling the original culture conditions. Migration rates were readout in the direction toward their respective SE (SE1, leftwards; SE2, rightwards; SE3, up-/downwards). LNCaP generally displayed significantly higher migration rates on plastic (8.1 ± 0.2 μm/h) compared with softer elastomer substrates. After expansion on the 1,600 kPa region, they showed up to 0.71-fold reduced migration rates toward the lymph node MTN (50 kPa) and up to 0.59-fold reduced migration rates toward prostate cancer mTME (90 kPa). Aligning with these findings, cells on the 1,600 kPa region showed no significant differences of migration rates compared with plastic. Consistent with this trend, PC3 migration rates were significantly higher on plastic (8.1 ± 0.3 μm/h) than on softer elastomer substrates. Migration rates on the starting SE3 (1,600 kPa) were 0.55-fold the migration rates compared with plastic and softer elastomer substrates. Relatively high migration rates were found toward bone marrow MTN (1 kPa, 0.67-fold) and prostate cancer mTME (90 kPa, 0.75-fold), indicating a pronounced migratory motivation toward the lower substrate stiffness. VCaP exhibited the lowest migration activity overall, with rates on plastic (0.9 ± 0.1 μm/h) and physio-elastic substrates (about 0.6 ± 0.1 μm/h), showing significantly reduced migration on the MTN-specific elasticities 1 and 1,600 kPa (see Figure 3D).

#### Physiologic MTN substrates diminish tumor cell invasion and cell-cell communication

For quantitative analysis of cell communication-facilitated invasion in response to substrate elasticities, we used a specially adapted Boyden Chamber to investigate interactions between tumor cells and healthy baiting-cells (see Figure 4A) under physio-elastic conditions. Healthy prostate epithelial cells (PNT2) served as baiting cells for LNCaP, and osteoblasts (MC3T3-E1) served as baiting cells for PC3 and VCaP.

**Figure 4:**
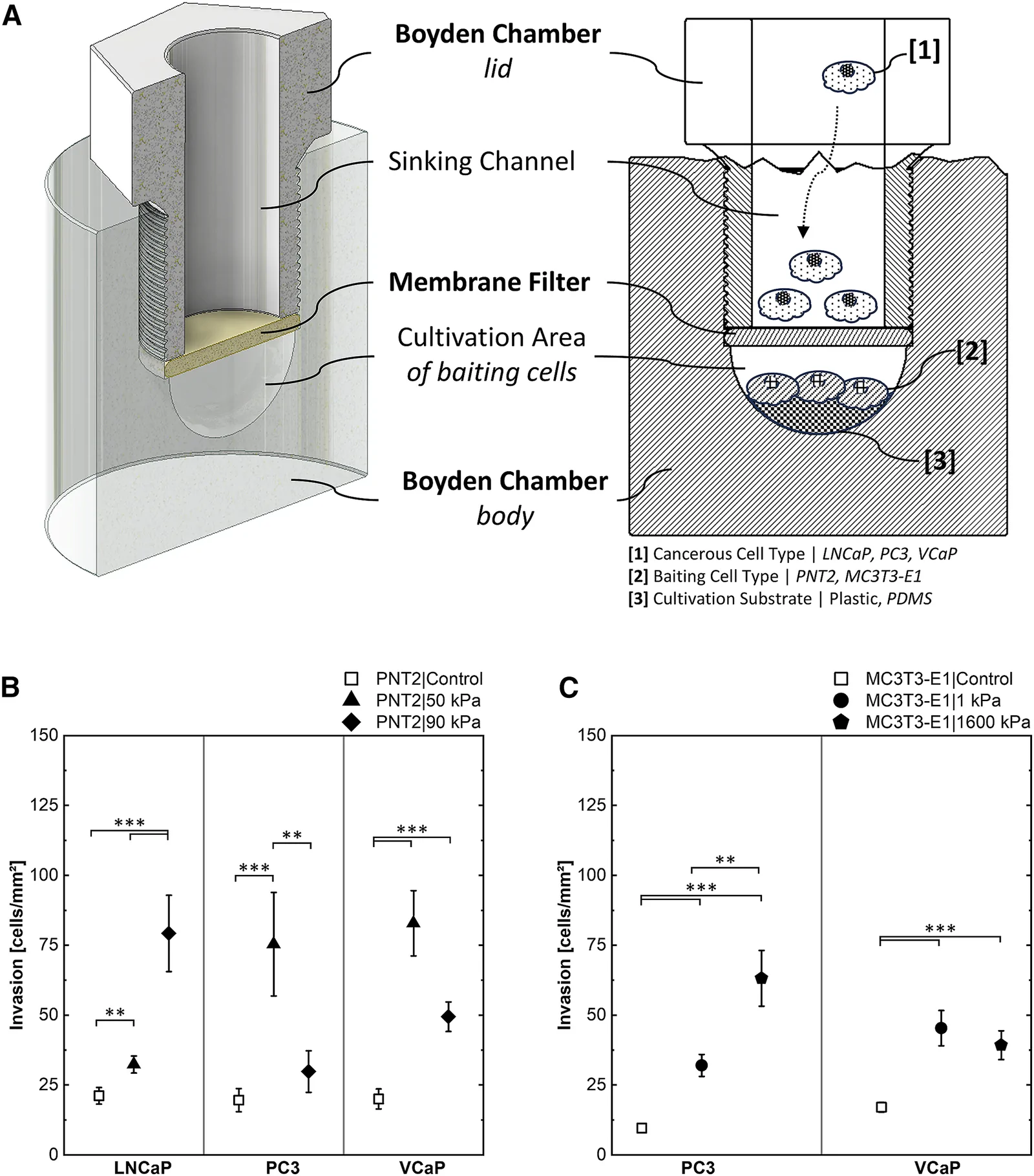
Effect of substrate stiffness of cultured baiting cells on prostate cancer cell invasion (A–C) In a specially designed Boyden Chamber, LNCaP, PC3, and VCaP were cultured as invading cells [1], separated by a membrane filter from healthy prostate epithelial cells (PNT2) and osteoblasts (MC3T3-E1) serving as baiting cells [2] (A). Baiting cells were cultured on plastic or mTME- and MTN-substrates [3]. Invasion rates for prostate cancer cell lines (LNCaP, PC3, and VCaP) were quantified after 4 h of invading by nuclei staining. Baiting cells PNT2 (B) and MC3T3-E1 (C) were cultured on either 50 kPa ▲, 90 Pa ◆ (PNT2/B) or 1 kPa, ● 1,600 kPa ⬟ (MC3T3-E1/C). Data are represented as mean ± SEM. Asterisks indicate significance levels determined by Mann-Whitney U test (MWU): ∗*p* ≤ 0.05, ∗∗*p* ≤ 0.01, and ∗∗∗*p* ≤ 0.001, based on at least three independent experiments.

When PNT2 were cultured on standard plastic substrates, all prostate cancer cell lines exhibited comparable baseline invasion rates of approximately 20 cells/mm^2^. However, when PNT2 was exposed to prostate tissue-specific healthy and mTME-elasticities, a significant increase in cell invasion was observed for all prostate cancer cell lines. Similarly, interactions between PC3 or VCaP and osteoblasts showed significantly increased invasion rates when osteoblasts were cultured on bone marrow (1 kPa) or trabecular bone substrates (1,600 kPa). On plastic, both cell lines showed invasion rates of 9–17 cells/mm^2^.

LNCaP displayed a 1.5-fold increase in invasion toward PNT2 cultured on 50 kPa, and up to a 3.8-fold increase when PNT2 were cultured on substrates with pathologic stiffnesses (90 kPa), as shown in Figure 4B. Interestingly, PC3 demonstrated their highest invasion rates in response to PNT2 cultured on 50 kPa, with invasion increasing by 3.9-fold compared with plastic controls. Toward PNT2 cultured on prostate cancer mTME substrates, PC3 showed a 1.5-fold increase in invasion rates (see Figure 4B). Similarly, VCaP also demonstrated their highest invasion rates in response to PNT2 cultured on 50 kPa with invasion increasing by 4.1-fold compared with plastic controls. Toward PNT2 cultured on prostate cancer mTME substrates, VCaP cells showed a 2.5-fold increase in invasion (see Figure 4B).

Notably, PC3 exhibited the strongest invasion toward baiting cells on substrates mimicking the physiological stiffness of trabecular bone, with 6.6-fold increased invasion rates compared with MC3T3-E1 cultured on plastic. Toward MC3T3-E1 cultured on 1 kPa, PC3 also showed significantly increased, yet lower, with 3.3-fold higher invasion rates (see Figure 4C). For VCaP, invasion rates peaked at 2.7-fold toward MC3T3-E1 cultured on substrates of 1 kPa elasticity (bone marrow), with only minor difference in invasion rates toward baiting cells cultured on 1,600 kPa (2.3-fold, see Figure 4C).

#### Modulation of the cytokine profile by culturing baiting cells on elastic substrates

To further deepen the understanding of the distinct invasion behavior of the prostate cancer cell lines in the Boyden Chamber experiments, differentially expressed cytokines of the baiting cell lines (PNT2 and MC3T3-E1) were quantified using a Cytokine-Array.

In PNT2, the use of physiologically inspired substrates resulted in a significant increase in the cytokines Ang-2, FGF, PlGF, and IL-6. On 50 kPa substrates, elevated levels of Ang-2 and IL-6 were particularly evident. In contrast, on 90 kPa substrates, FGF and PlGF emerged as the predominantly altered cytokines. These findings indicate that distinct matrix stiffnesses elicit specific cytokine responses in PNT2. Compared with cytokine secretion on plastic substrates, an increased secretion of Ang-2 (3.7-fold on 50 kPa, 3.2-fold on 90 kPa) and FGF (1.3-fold on prostate cancer mTME), and IL-6 (1.5-fold on 50 kPa and 1.1-fold on 90 kPa) was detected (see Figures 5A and 5B). Analysis of the temporal cytokine secretion profiles under different physio-elastic culture conditions revealed distinct substrate-dependent patterns. After 48 h of cultivation, the secretion levels of IL-6, FGF, CXCL8, PECAM-1, PlGF, and TNFα were highest in cells cultured physio-elastic substrates compared with plastic controls. In contrast, Ang-1 secretion reached its maximum after 72 h and remained elevated on physio-elastic substrates relative to plastic cultures. VEGF displayed a distinct temporal profile, with an initial secretion peak observed after 24 h on physio-elastic substrates. However, VEGF levels subsequently declined during prolonged culture, whereas secretion remained elevated on plastic controls, ultimately resulting in higher VEGF concentrations under conventional culture conditions at later time points (see Table S1).

**Figure 5:**
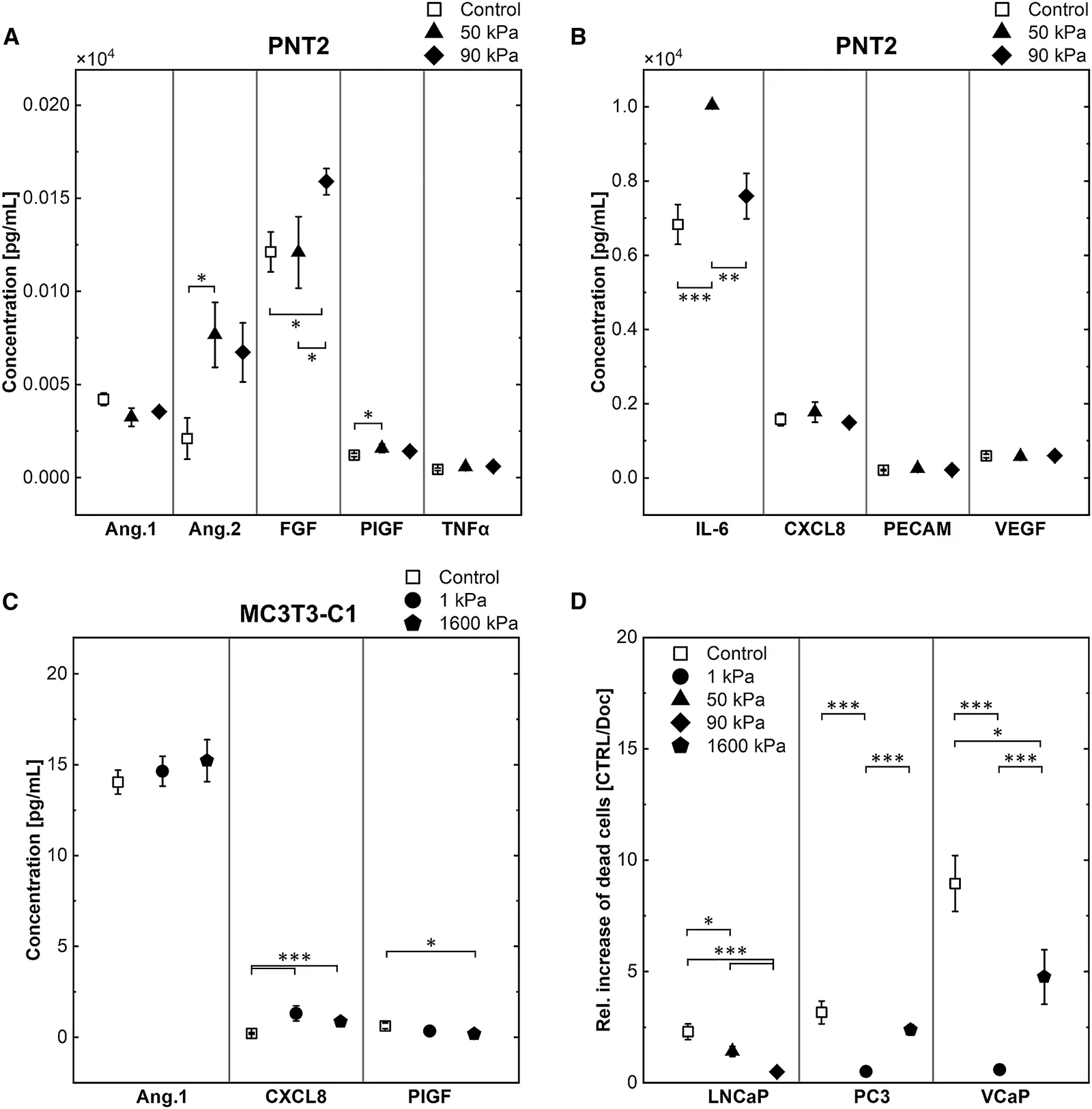
Influence of substrate elasticity on cell communication and on therapy sensitivity (A–C) The cytokine secretion induced by different culture conditions during cultivation in the specially designed Boyden Chamber (see Figure 4A) was quantified using the LEGENDplex Cytokine Array. Distinct cytokine patterns were identified for PNT2 (A and B) and MC3T3-E1 (C) depending on the culture conditions. All detected cytokines, including angiopoietin (Ang.) 1/2, fibroblast growth factor (FGF), interleukin-8 (CXCL8), interleukin (IL)-6, placental growth factor (PlGF), tumor necrosis factor alpha (TNFa), platelet endothelial cell adhesion molecule (PECAM), and vascular endothelial growth factor (VEGF) are displayed. Prostate cancer cell lines LNCaP, PC3, and VCaP were treated with the chemotherapeutic agent docetaxel for 48 h (D). LNCaP were cultured on 50 kPa substrates ▲ or 90 kPa ◆, while PC3 and VCaP were cultured on substrates with elasticities of 1 kPa , ● and 1,600 kPa ⬟. The increase in cell death upon treatment was measured using LIVE/DEAD Fixable Blue Dead Cell Stain Kit and normalized relative to an untreated control for each respective culture condition (Doc/CTRL). Data are represented as mean ± SEM. Asterisks indicate significance levels determined by Mann-Whitney U test (MWU): ∗*p* ≤ 0.05, ∗∗*p* ≤ 0.01, and ∗∗∗*p* ≤ 0.001, based on at least three independent experiments.

In the case of MC3T3-E1, Ang-1 emerged as the predominant factor, remaining consistently dominant across all substrate elasticities. Still, an altered cytokine pattern (see Figure 5C), as well as a significant increase in the cytokine CXCL8 (IL8) was observed on the physiological substrates with a 6.1-fold increase at 1 kPa and a 4.0-fold increase at 1,600 kPa compared with plastic. Ang-2 exhibited an early secretion peak after 24 h on physio-elastic substrates and remained elevated under these conditions. After 48 h, CXCL8 secretion reached its maximum on physio-elastic substrates, whereas the highest PlGF levels were detected in cells cultured on plastic controls. After 72 h, Ang-1 secretion peaked in plastic-cultured cells (see Table S2). These findings demonstrate that substrate elasticity not only modulates the magnitude of cytokine secretion but also influences the temporal dynamics of pro-inflammatory and pro-angiogenic signaling factors.

#### Cell lines cultured on mTME show reduced treatment-sensitivity

To investigate the influence of mTME on the chemosensitivity of the prostate cancer cell lines, treatment with the chemotherapeutic agent docetaxel was conducted, and cell viability was quantified (see Figure 5D). To verify comparable bioavailability of the chemotherapeutic agent, corresponding controls were carried out on plastic and physio-elastic substrates (see Figure S3A). To investigate whether substrate mechanics influence chemotherapeutic responsiveness, LNCaP cells cultured on physio-elastic substrates were exposed to increasing concentrations of docetaxel (0.1–10 nM), and chemosensitivity was assessed by quantifying dead cells. While only minor differences were observed at lower docetaxel concentrations, a trend toward reduced chemosensitivity became apparent on physio-elastic substrates (90 kPa) at concentrations ≥ 1 nM. This effect reached significance at 10 nM docetaxel, where substrate stiffness markedly influenced treatment response (see Figure S3B). Specifically, cells cultured on 50 kPa substrates exhibited a significant reduction in docetaxel-induced cell death with 39 % fewer dead cells compared with plastic control. An even more pronounced effect was observed on 90 kPa substrates, where the number of dead cells was reduced by approximately 80 % relative to plastic-cultured cells. The examination of PC3 revealed the greatest reductions in sensitivity to docetaxel on 1 kPa substrates, with cell death rates reduced by 84 % compared with cell death on plastic control. Significant reductions were also observed on 1,600 kPa substrates, with cell death rates reduced by 25 % compared with cell death in the plastic cultures (see Figure 5D). Similarly, the examination of VCaP revealed the greatest reductions in sensitivity to docetaxel on 1 kPa substrates, with cell death rates reduced by 93 % compared with cell death on plastic controls. Additionally, significant reductions also were observed at 1,600 kPa substrates, with cell death rates reduced by 47 % compared with plastic cultures (see Figure 5D). In addition to the chemosensitivity studies, the androgen receptor antagonist enzalutamide was evaluated to determine whether physiologically relevant substrate mechanics influence therapeutic responsiveness. Like the effects observed with docetaxel, physio-elastic substrates significantly altered the cellular response to enzalutamide treatment. Notably, cells cultured on 90 kPa substrates exhibited significantly reduced sensitivity to enzalutamide across the entire concentration range tested (0.1–10 μM) compared with cells cultivated on controls. These findings indicate that physio-elastic substrate mechanics can modulate not only chemotherapeutic efficacy but also responsiveness to androgen receptor-targeted therapies (see Figure S3C).

### Cell-matrix interactions on structured hydrogels increase with substrate elasticity

We investigated the effect of substrate elasticity and topography using 2.5D-PEGDA-GMA hydrogel patches with structured surfaces. Hydrogels featuring hexagonal topographies and with E_eff_ of 40 kPa (15 wt.%), representing the benign prostate tissue, and 115 kPa (20 wt.%), representing the prostate cancer mTME, were fabricated. The selection of a hexagonal pattern was chosen to represent the large voids and dense areas known from histological surgery specimens (see Figure S1).

#### Cell morphology

The results showed a pronounced change in morphology parameters on structured prostate cancer mTME hydrogels (see Figures 6D–6F). Cell roundness decreased significantly for LNCaP and PC3 from softer to stiffer structured hydrogels. VCaP showed an adverse effect with decreased roundness on softer hydrogels.

**Figure 6:**
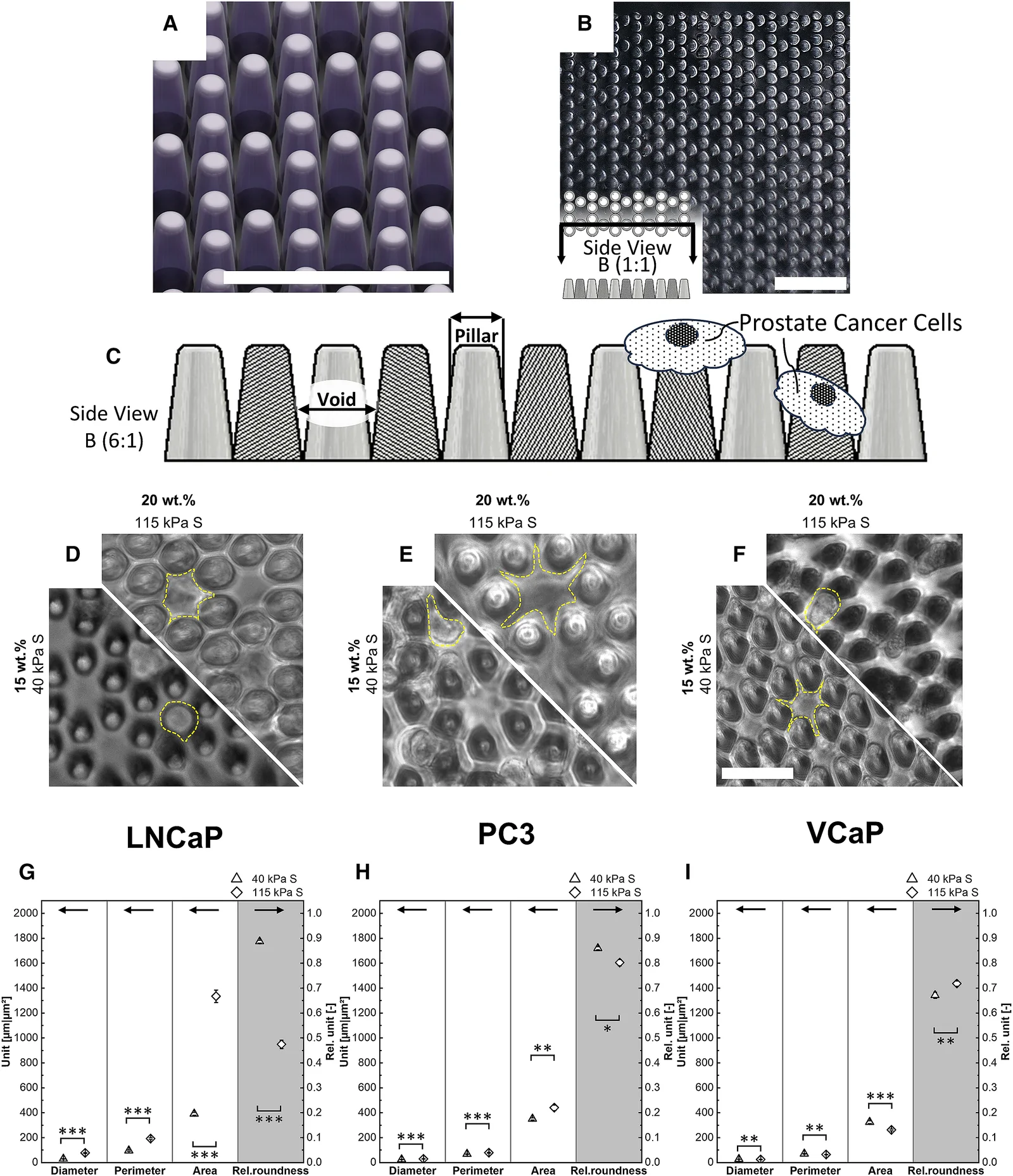
Properties of hydrogel patches and their influence on cell morphology (A–I) The topological features are conical pillars, arranged in a hexagonal pattern. Six pillars circle around one hole (void): (A) computer rendering, (B) microscopy image, and (C) technical drawing. The technical drawings view in (B) (B 1:1) bottom left, and (C) (B 6:1) show the alternating pillar pattern in one plane perpendicular to the microscopies top view of B. Hatched structures are cut by the views plane. The morphological characteristics of the prostate cancer cell lines on different hydrogel patch elasticities and topographies (structured (S) 15 wt.% △/△ and 20 wt.% ◊/◊) are depicted in (D–F). Cell morphology analysis of diameter, perimeter, area (G–I, left *y* axis) and level of roundness (G–I, right *y* axis) were analyzed. A roundness value of 1.0 represents an ideal circular shape. Data are represented as mean ± SEM. Asterisks indicate significance levels determined by Mann-Whitney U test (MWU): ∗*p* ≤ 0.05, ∗∗*p* ≤ 0.01, and ∗∗∗*p* ≤ 0.001 based on at least 100 cells. Scale bars, 100 μm.

On the stiffer, structured prostate cancer mTME hydrogel patches, LNCaP showed the largest geometric increases (diameter: 2.7-fold, perimeter: 2.0-fold, area: 3.4-fold), leading to the greatest reduction of 47 % to below 0.5 relative units of roundness. PC3 displayed the same behavior, yet less pronounced. The changes in morphology (diameter, 1.2-fold; perimeter, 1.2-fold; area, 1.3-fold) lead to approximately 10 % decrease (see Figures 6G–6I) in relative roundness on stiffer substrates compared with soft hydrogels. VCaP showed a contrary response to the structured prostate cancer mTME hydrogels with reduced geometric characteristics on stiffer substrates compared with soft hydrogels (diameter, 0.9-fold; perimeter, 0.9-fold; area, 0.8-fold). Interestingly, unlike flat PDMS surfaces, structured hydrogels altered VCaP circularity, with softer hydrogels leading to decreased cell roundness.

#### Cellular cytoskeleton

The characterization of the cytoskeletal components, actin and paxillin, is shown in Figures 7A–7C as immunofluorescence staining. On the structured prostate cancer mTME hydrogels, increased stiffness resulted in a significant upregulation of actin (1.48-fold) and non-significant upregulation of paxillin (1.05-fold) for LNCaP. On stiffer, structured prostate cancer mTME hydrogels, PC3 displayed a significant increase in actin (11.49-fold) and 1.84-fold increase in paxillin expression, contrary to the switch of actin to increased paxillin expression from soft to stiffer PDMS surfaces. For VCaP, on structured hydrogels, these changes were modest, with actin showing a non-significant 1.02-fold increase and paxillin a significant 1.41-fold increase (see Figure 7D). Comparable alterations in actin filament orientation were also observed in LNCaP and PC3, whereas VCaP did not exhibit any significant changes in protein alignment for this analysis (see Figure 7E).

**Figure 7:**
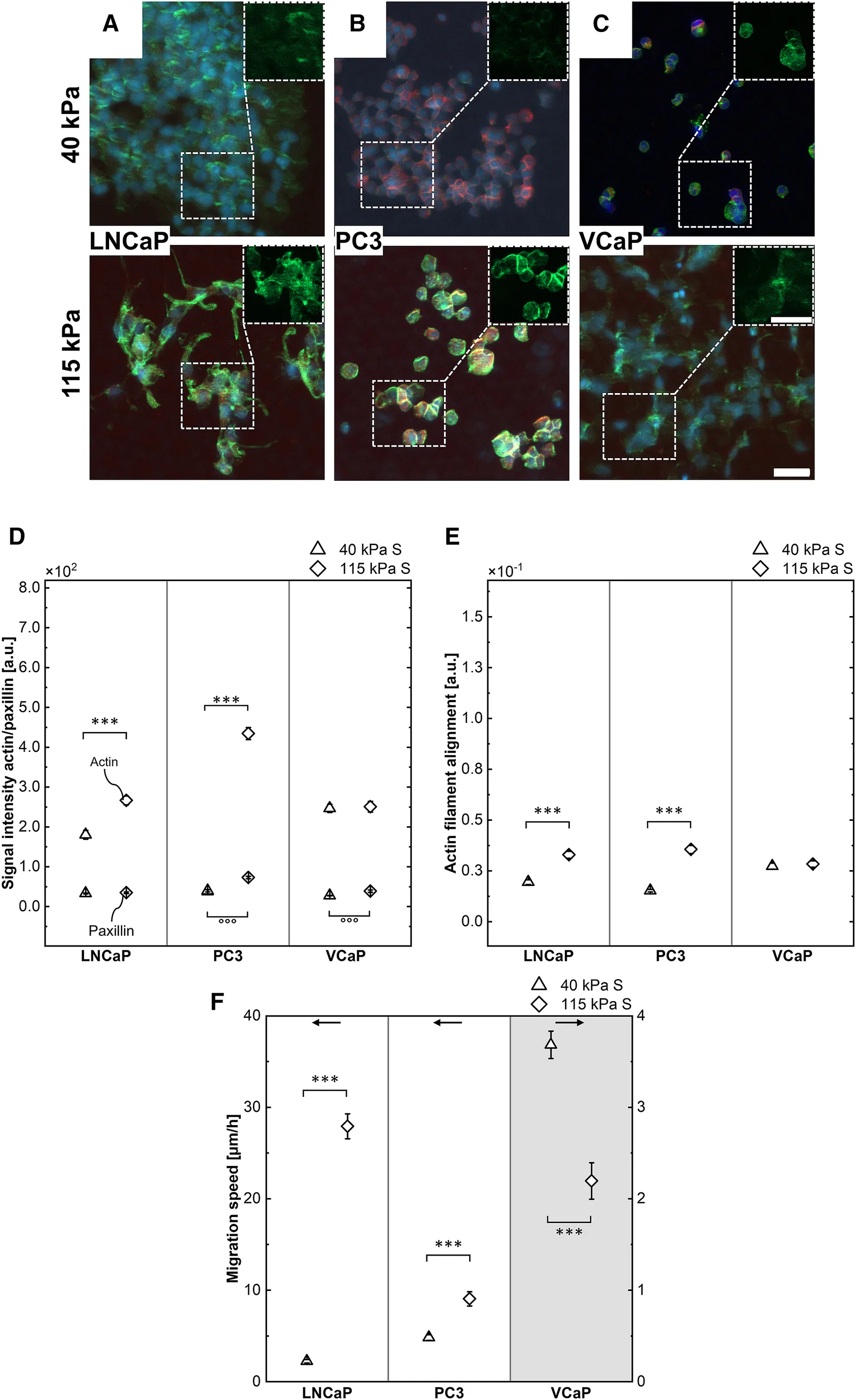
Effect of topography and elasticity on prostate cancer cell morphology, cytoskeleton components, and migrative activity (A–C) The corresponding cytoskeleton protein expression of actin (green), paxillin (red), and nuclei (blue) for LNCaP (A), PC3 (B), and VCaP (C) on structured prostate cancer mTME hydrogel patches are depicted. The intensity of actin is represented by hollow symbols, while paxillin (red) is indicated with an additional dot. (D–F) In addition, a comparative characterization of actin filament orientation (E) and cell migration speed within the hydrogels (F) was performed. Asterisks indicate significance levels of actin while circles indicate significance levels for paxillin with *p* ≤ 0.05 (∗/○), *p* ≤ 0.01 (∗∗/○○), and *p* ≤ 0.001 (∗∗∗/○○○), based on at least 100 cells. (D and E) The observed migrative activities of the respective cell lines are depicted in (C). Data are represented as mean ± SEM. Asterisks indicate significance levels determined by Mann-Whitney U test (MWU): ∗*p* ≤ 0.05, ∗∗*p* ≤ 0.01, and ∗∗∗*p* ≤ 0.001, based on at least 60 cells. (F) Scale bars, 20 μm; insert scale bars, 20 μm.

#### Migration

Live cell tracking revealed significant alterations in migratory behavior based on substrate stiffness and topography (see Figure 7F). Migration on hydrogel substrates was generally significantly faster compared with PDMS controls (see Figure 3D), with velocities increasing up to 6.5-fold (see Figure 3D).

On the structured prostate cancer mTME hydrogels, increased stiffness enhanced migration for all prostate cancer cell lines. LNCaP cells exhibited the most pronounced response, with a 12.50-fold increase in velocity, followed by PC3.

### Human prostate cancer isolates keep translational tumor marker-expression

To further investigate how substrate elasticity influences cell behavior, primary prostate cancer cell isolates (Gleason 7b, Gleason 10) were cultured on mTME substrates (90 kPa) or non-physiological standard culture plates and analyzed regarding morphological changes and expression of prostate cancer-specific markers. These results are shown in Figure S4.

#### Phenotypical change in cell morphologies and proliferation

After 24 h of cultivation, cells from Gleason 7b tissue isolates attached individually to standard substrates, whereas on mTME cells predominantly formed smaller aggregates (see Figure S4A). At 120 and 168 h, these differences became less pronounced, with both substrates exhibiting elongated, loosely distributed cells. Notably, more matrix and cell clusters were observed on control cultures (see Figure S4B). By 240 h, prior to termination of the experiments and qRT-PCR analysis of tumor-specific markers, cell density had increased more pronounced on physio-elastic cultures, and morphological differences to control cultures were evident (see Figures S4C and S4D). At 24 h of culture, the Gleason 10 isolates displayed a more heterogeneous pattern. Both substrates were densely populated with large cell clusters observed on non-physiological control cultures and, presumably, a higher general adherence compared with physio-elastic substrates (see Figure S4E). By 120 h, mTME cultures exhibited almost no cell clusters compared with standard cultures. On both substrates, elongated cells were present, distributed loosely on physiological mTME cultures and primarily near cell clusters on control cultures (see Figure S4F). After 168 h, cell morphology and distribution on mTME substrates remained largely unchanged. In contrast, there was a clear increase in cell numbers, presumably originating from cell clusters on control cultures. Moreover, the morphology of these cells shifted from elongated to more rounded shapes (see Figure S4G). By 240 h, confluence further increased on control substrates, with only a minority of cells retaining elongated shapes, while an increase in cell roundness and a reduction in elongation on physio-elastic samples was also observable (see Figure 4H).

#### Expression pattern of relevant tumor markers

To quantify the amount of prostate cancer-specific tumor markers after culturing cells on mTME or non-physiological standard cultures for 10 days, a qRT-PCR analysis for androgen receptor (AR), prostate cancer antigen 3 (PCA3), and prostate-specific membrane antigen (PSMA) was performed (see Figure S5A). The significant higher expression of AR and PCA3 for cultivation on mTME substrates compared with non-physiological control samples was observed for both Gleason score isolations. For PSMA, only the Gleason 7b isolation showed significantly higher expression. Interestingly, the Gleason 10 isolation expressed higher amounts of the other tumor markers on physio-elastic conditions. Immunohistochemical staining of the original patient tissues for AR and PSMA further demonstrated that physio-elastic substrate conditions preserved expression patterns closely resembling those of the corresponding native tissues. Specifically, cultures derived from the Gleason 10 patient exhibited a 1.62-fold higher proportion of AR-positive cells compared with cultures derived from the Gleason 7b patient, closely matching the expression profile observed in the original tissue specimens, where AR expression was 1.71-fold higher in the Gleason 10 specimen. Similarly, PSMA staining in the physio-elastic cultures revealed a 0.63-fold reduction in expression in the Gleason 10-derived cultures relative to the Gleason 7b-derived cultures, which was in strong agreement with the expression pattern of the corresponding patient tissues, where PSMA levels were reduced by 0.83-fold in the Gleason 10 specimen. Collectively, these findings indicate that the physiologically relevant culture conditions can preserve patient-associated molecular characteristics and maintain relevant tumor marker expression *ex vivo* (see Figures S5B–S5D).

## Discussion

### Relevance of substrate elasticity and topography on prostate cancer cell lines

Our findings demonstrate that both substrate elasticity and defined topographies modulate prostate cancer cell behavior and, importantly, the interactions between malignant and non-malignant counterparts. These observations are consistent with physiological *in vivo* conditions, where altered metabolic activity and cytoskeletal remodeling shape tumor progression.

Studies on hepatocellular carcinoma have demonstrated that hydrogels, when adapted to stiffer, more physio-elastic conditions, lead to increased glucose consumption and lactate production.^34^ In line with these findings, we observed significantly upregulated LDH activity in prostate cancer cell lines (LNCaP and PC3) cultured on prostate cancer-specific PDMS substrates compared with traditional cell culture substrates (see Figure 1). Typically, elevated LDH activity in benign cells *in vivo* indicates cellular stress, such as that induced by hypoxia.^35^^,^^36^ However, tumor cells not only utilize LDH but also preferentially rely on it even under aerobic conditions.^37^^,^^38^ Our observed increase in LDH activity in prostate cancer cell lines suggests that physiological substrate elasticity may facilitate a more tumor-specific metabolic shift. The simultaneous increase in tumor cell proliferation further supports this hypothesis by ruling out the possibility of degenerative behavior typically associated with cellular stress. The absence of a significant proliferative response in VCaP cells may be related to their characteristic TMPRSS2-ERG fusion status. This genomic alteration profoundly reshapes the transcriptional landscape of VCaP cells and has been associated with activation of the NOTCH signaling pathway. Previous studies have demonstrated that ERG can directly regulate the expression of NOTCH pathway components, including NOTCH1 and HES1, thereby promoting oncogenic signaling programs. Consequently, the cellular response to elastomer-induced microenvironmental cues may be attenuated by the dominant influence of constitutively active ERG-driven signaling networks.^39^^,^^40^ Similarly, the reduction in actin intensity and alignment observed in our models aligns with structural patterns identified in tissue sections, highlighting the physiological relevance of cytoskeletal reorganization (compare Figures S1, S2, and S7). The correlation between increased substrate elasticity and larger cell areas, decreased roundness, and pronounced cytoskeletal reorganization in our results is consistent with the findings of other studies, i.e., in liver cancer, in which adaptation to the physio-elastic stiffness of liver cirrhosis led to similar changes.^41^ Given the central role of the cytoskeleton in cellular mobility and migration, it is expected that morphological changes, which are inherently linked to alterations in the cytoskeleton, significantly impact migratory activity and vice versa. The observation of this migratory behavior may not only provide insights into disease progression but also may offer clues about its metastatic potential.^42^ Our investigation into cytoskeletal organization and altered paxillin expression supports this expectation, although no distinct pattern of increased or decreased protein expression was directly linked to migratory activity. Nonetheless, we observed a decrease in migratory behavior across all three cell lines as they approached the stiffness typical of metastatic target regions. When incorporating aspects of a more physiological environment through increased dimensionality using structured PEGDA-GMA hydrogel patches, the same migratory behavior was observed, but with significantly higher migratory activity on the prostate cancer-specific stiffness. While these experiments lack comparison with 1 and 1,600 kPa elasticities due to material system limitations, the importance of introducing topographies or three-dimensionality to modulate migration is evident.^43^^,^^44^ Such findings align with efforts to investigate cell migration under chemotactic migration through 3D constraints, as demonstrated in microfluidic perfusion culture systems designed to mimic confined microenvironments.^45^ Three-dimensional spheroid, organoid, and hydrogel-encapsulation models provide additional levels of cellular organization and cell-matrix interaction that are not fully captured by 2D or 2.5D substrates. Such systems can reproduce spatial constraints, cell-cell contacts, and three-dimensional matrix interactions and may therefore reveal phenotypic responses that differ from those observed on planar substrates. In particular, encapsulation of prostate cancer cells within stiffness-matched hydrogels could further influence migration, cytoskeletal organization, invasion, and therapeutic responsiveness. Whether these additional dimensions would amplify the elasticity-dependent effects observed in our study remains to be determined experimentally. The 2.5D approach used here therefore represents an intermediate level of microenvironmental complexity that enables controlled investigation of elasticity and topography while retaining accessibility for imaging and quantitative analysis. Future studies combining stiffness-matched 3D matrices with patient-derived prostate cancer cells may help determine how these mechanical cues interact with three-dimensional architecture and cellular heterogeneity.

Most notably, we observed significant reductions in docetaxel sensitivity under specific mechanical conditions (see Figure 5). This finding echoes previous reports showing that standard 2D cultures often overestimate drug efficacy, whereas microenvironment-mimicking systems reveal reduced responsiveness that better reflects *in vivo* data.^46^ The potential for therapeutic cancer treatments to cause significant damage to healthy tissues underscores the importance of tailoring treatment to each patient’s condition, minimizing harm, and improving individual prognosis. Studies using 3D models have shown that ECM elasticity influences cellular responses to chemotherapeutics and can contribute to chemoresistance.^13^^,^^47^ Additionally, tumor-specific metabolic shifts, including increased LDH activity in the context of microenvironmental acidification and nutrient deprivation, may further compromise therapeutic efficacy.^38^^,^^48^ Similar to ductal adenocarcinoma, where substrate elasticity alters chemotherapeutic responses,^49^ we observed a marked reduction in docetaxel sensitivity in all three prostate cancer cell lines. This suggests that similar effects may occur in other cancers, emphasizing the need for physiologically relevant elastic substrates when evaluating drug responses in preclinical *in vitro* models. Moreover, modern therapeutic approaches, such as CAR-T cell therapies, are significantly influenced by the mechanical properties of tumor cells. Studies have demonstrated that increased tumor cell stiffness induced, for example, by cholesterol integration into the membrane can reduce T-cell activation.^50^ These findings highlight the important role of biomechanical properties in both therapeutic efficacy and the preclinical evaluation of tumor cells. Understanding these mechanical factors is therefore essential for optimizing immunotherapeutic strategies and advancing precision oncology.

These adaptive changes are not limited to cancerous cell lines; healthy cells such as MC3T3-E1 and PNT2 also show pronounced alterations in chemokine release (see Figures 4 and 5) as a response to the TME created by cancerous cells during their upregulated and unsustainable proliferation and metabolism (compare Figure 1). In the context of metastasis, cellular signaling molecules, particularly cytokines, are frequently implicated in cancer progression. The release of cytokines and chemokines facilitates the multistep process of tumor development by promoting cell proliferation, migration, and ECM remodeling.^37^^,^^48^^,^^51^^,^^52^ Increased expression of growth factors such as Ang-1, Ang-2, VEGF, and FGF has been observed in metastatic prostate cancer cell lines, enhancing cell growth, differentiation, proliferation, and angiogenesis.^51^^,^^53^ Furthermore, inhibition of IL-6 in PC3 prevents cell invasion, whereas overexpression of CXCL8 (IL-8) stimulates tumor growth and cell cycle progression.^52^ Notably, CXCL8 emerged as a prominent component within the present model and warrants particular attention given its well-established role in prostate cancer progression and bone metastasis. Elevated CXCL8 signaling has been associated with enhanced tumor cell migration, invasion, and proliferation, as well as with the establishment of a permissive metastatic niche within the bone microenvironment. The increased CXCL8 expression observed in our system therefore suggests that the elastomer-based co-culture model captures aspects of tumor-stroma interactions associated with the metastatic cascade. Importantly, CXCL8-mediated signaling represents a critical communication axis between prostate cancer cells and bone-associated cellular compartments, contributing to tumor cell homing, colonization, and subsequent outgrowth at metastatic sites.^54^ Joining prostate cancer, other cancers, i.e., mammary carcinoma, find favorable metastasis sites in the cytokine-rich microenvironment of bone, leading to promoted organotrophic metastasis.^51^^,^^55^^,^^56^ In our experiments, we observed an evident increase in cytokine and growth factor release in the adapted Boyden Chamber experiments, indicating that microenvironmental elasticity influences cytokine and growth factor signaling associated with angiogenic activity. Notably, the significant increase in Ang-2 and FGF release, compared with the pronounced upregulation of IL-6, highlights a reinforced proliferative response, even in healthy baiting cell lines. This suggests that the correlation between substrate stiffness and cytokine release is not limited to malignant cell types and severely influences MTN composition. Based on these findings, it is crucial that the influence of substrate elasticity is always considered when assessing potential metastatic regions and their invasive potential *in vitro*.

These peculiarities underscore the inherent limitations of *in vitro* models based solely on flat, rigid standard culture vessels, which typically exhibit elasticities in the gigapascal range. This influence of substrate elasticity on the outcomes of *in vitro* assays remains insufficiently addressed, often leading to compromised results.

### Future perspectives of *in vitro* models for prostate cancer progression

Advancing *in vitro* methodology necessitates the precise replication of physiological microenvironments, accounting not only for chemical composition but also for mechanical properties and dynamic stimulation. A comprehensive understanding of both chemical and mechanical signal transduction pathways is critical to elucidating their roles in disease progression. The interplay between elastic and topographic cues in shaping cellular behavior, facilitated by advanced assay systems, may help elucidate the mechanisms underlying disease progression.

Besides this, suggested control over cancer-specific behavior *in vitro*, primary prostate cancer cell isolates culture lacks the robustness and selectivity toward malignant cell types for further investigation. As our preliminary results of such primary cells isolate culture already promisingly indicate, the usage of such mTME-specific substrates may support the development of more robust long-term *in vitro* culture approaches. These efforts will provide a foundation for translational applications, bridging *in vitro* discoveries to *in vivo* models and ultimately driving the development of innovative clinical interventions.

### Limitations of the study

Several limitations should be considered when interpreting our findings. First, most experiments were performed using immortalized prostate cancer cell lines. These cells have undergone extensive adaptation to *in vitro* culture and may therefore not fully reflect the heterogeneous responses of primary tumor cells to substrate elasticity and topography. Although our preliminary experiments with primary prostate cancer cells showed preserved cancer-associated expression profiles and increased viability on mechanically tumor-relevant substrates (Figures S4 and S5), primary cell cultures remain challenging to establish and maintain. Prostate cancer cultures commonly exhibit progressive loss of tumor-specific characteristics during extended culture, which may result from the preferential expansion of non-malignant cells within heterogeneous tumor samples. Establishing conditions that selectively preserve malignant cell populations therefore remains an important challenge. Increasing the initial tumor cell density and promoting sustained proliferative activity may help minimize competitive expansion of non-malignant cells and improve the robustness of long-term primary cultures.^57^^,^^58^ Second, the models used in this study primarily address substrate elasticity and selected topographical features. The native tumor microenvironment is considerably more complex and includes additional biophysical and biochemical parameters, such as ECM composition, three-dimensional architecture, interstitial pressure, fluid flow, and oxygen availability. In particular, incorporating physiologically relevant oxygenation and more complex three-dimensional topographies will be important to determine whether the observed elasticity-dependent phenotypes are maintained under increasingly physiological conditions.

Finally, although changes in migration, cytokine expression, cytoskeletal organization, docetaxel, and enzalutamide responsiveness were associated with substrate elasticity, the underlying molecular mechanisms were not comprehensively resolved in this study. Further investigation using mechanistic perturbation approaches will be required to establish how specific mechanotransduction pathways connect extracellular elasticity to these functional responses. Thus, while the present models provide reproducible systems for investigating mechanical regulation of prostate cancer cell behavior, their interpretation should account for the biological and biophysical simplifications inherent to these *in vitro* platforms.

## Resource availability

### Lead contact

Requests for further information and resources should be directed to and will be fulfilled by the lead contact, Marco Hoffmann (marchoffmann@ukaachen.de).

### Materials availability

Physiological substrates presented in this study will be made available upon request. Provision of materials may require payment and/or completion of a materials transfer agreement, particularly, where potential commercial applications exist.

### Data and code availability

• This study did not generate or use any standardized data types requiring deposition in a data-type-specific repository. All other data supporting the findings of this study are available from the lead contact upon reasonable request.
• This study does not report original code.
• Any additional information required to reanalyze the data reported in this paper is available from the lead contact upon reasonable request.

## Acknowledgments

We thank Kerstin Junker (Department of Urology and Pediatric Urology, Saarland University, Homburg/Saar, Germany) for generously providing LNCaP and VCaP cells and Malin Hagberg Thulin (Department of Internal Medicine and Clinical Nutrition, University of Gothenburg) for providing MC3T3-E1. We sincerely thank Danny David Jonigk, Director of the Institute of Pathology at RWTH Aachen University Hospital, and his team for the preparation of control sections for receptor-staining of the patient tissue specimens, as well as for the pathological evaluation and annotation of the relevant tumor regions.

## Author contributions

Conceptualization, M.H., R.P., and M.S.; methodology and investigation, M.H., R.P., A. Hermann, S.G., T.F.E, M.B., R.A., A. Honné, J.P.M., J.K.B., and P.F.; writing – original draft, M.H., R.P., and M.S.; writing – review and editing, M.H., R.P., S.G., P.B., and M.S.; funding acquisition, M.H. and M.S.; resources, M.W. and M.S.; supervision, M.H., J.K., M.W., and M.S.

## Declaration of interests

The authors declare no potential conflicts of interest.

## Declaration of generative AI and AI-assisted technologies in the writing process

During the preparation of this work, the authors used generative artificial intelligence (ChatGPT-4.o) to assess the understandability and accessibility of the manuscript. After using this tool or service, the author(s) reviewed and edited the content as needed and take full responsibility for the content of the publication.

## STAR★Methods

### Key resources table

**Table:** 

REAGENT or RESOURCE	SOURCE	IDENTIFIER
**Antibodies**		

Paxillin Monoclonal Antibody	Thermo Scientific	#AHO0492 RRID: AB_2536312
Goat anti-mouse IgG (H+L) Alexa Fluor^TM^ (568 nm)	Thermo Scientific	A-11004 RRID: AB_2534072
Phalloidin Alexa Fluor (488 nm)	Thermo Scientific	A12379 RRID: AB_2315147
Hoechst 33342 Ready Flow	Thermo Scientific	R37165 RRID: n.a.
PSMA / FOLH1 Antibody	NSJ Bioreagents	F43183, LOT: 110622bw RRID: n.a.
Androgen receptor Antibody	Merck	06–680 RRID: AB_310214
Goat anti-rabbit IgG (H+L) Alexa Fluor^TM^ (488 nm)	Thermo Scientific	A-11008 RRID: AB_143165

**Chemicals, peptides, and recombinant proteins**		

Fetal bovine serum superior (FBS)	Merck	S0615
Human fibronectin	Merck	FN0635
Sylgard 184 PDMS	VWR/DOW	DOWC25100445
PEGDA (575 Da)	Merck	455008
GMA	Merck	779342
LAP photoinitiator	Merck	900889
hESC StemPro medium	Thermo Scientific	A1000701
R1881	Merck	R0908
Y-27632	Merck	Y0503-5MG
Human bFGF	Thermo Scientific	PHG0264
Docetaxel	Selleckchem	S1148
Enzalutamide (MDV3100)	Selleckchem	S1250

**Critical commercial assays**		

CyQuant™ LDH Cytotoxicity Assay Kit	Thermo Scientific	C20300
LIVE/DEAD™ Viability/Cytotoxicity Kit	Thermo Scientific	L3224
LEGENDplex™ Human Angiogenesis Panel 1	BioLegend	741215
Human Tumor Dissociation Kit	Milteny Biotec	130-095-929
MycoSPY® PCR Kit	Biontex	M020-50

**Experimental models: Cell lines**		

LNCaP (human prostate cancer cell line, lymph node metastasis)	Merck	89110211, RRID: CVCL_0395
PC3 (human prostate cancer cell line, bone metastasis)	ATCC	CR-1435, RRID: CVCL_C3M5
VCaP (human prostate cancer cell line, vertebral bone metastasis)	Merck	06020201, RRID: CVCL_C0QQ
PNT2 (human prostate epithelial cell line)	Merck	95012613, RRID: CVCL_2164
MC3T3-E1 (murine osteoblast cell line)	ATTC	CRL-2593, RRID: CVCL_0409

**Experimental models: Organisms/strains**		

Primary cells from Gleason Score 7b and Gleason Score 9 (human)	University Hospital Aachen, Department of Urology and Pediatric Urology	Ethics approval: EK23-051
FFPE benign and cancer prostate tissue sections (pathologically confirmed patient material)	University Hospital Aachen, Department of Urology and Pediatric Urology	Ethics approval: EK25-023

**Oligonucleotides**		

TaqMan Androgen Receptor (AR) Assay	Thermo Scientific	Hs00171172_m1
TaqMan Prostate Cancer Antigen 3 (PCA3) Assay	Thermo Scientific	Hs01371939_g1
TaqMan Antigen (PSMA/FOLH1) Assay	Thermo Scientific	Hs00379515_m1
TaqMan Glyceraldehyde 3-phosphate dehydrogenase (GAPDH) Assay	Thermo Scientific	Hs02786624_g1

**Software and algorithms**		

ImageJ	NIH	RRID:SCR_003070
FibrilTool plugin	ImageJ plugin	RRID:SCR_016773
Zen3.8	Zeiss	RRID:SCR_013672
Real Statistics Resource Pack	Real Statistics	Release 9.6.1
Origin 2019	OriginLab	Version 2019
Microsoft Excel 2019	Microsoft	RRID:SCR_016137
Blender	Blender Foundation	Version 3.1
Inventor Professional	Autodesk	Version 2023
LEGENDplex Data Analysis Software	BioLegend	Version 2023–02–15
StepOne Software	Thermo Scientific	Version 2.3

### Experimental model and study participant details

#### Human prostate cancer cell lines

Three human prostate cancer cell lines were used in this study: LNCaP (Merck, 89110211, RRID: CVCL_0395), PC3 (ATCC, CR-1435, RRID: CVCL_C3M5), and VCaP (Merck, 06020201, RRID: CVCL_C0QQ). A healthy human epithelial prostate cell line, PNT2 (Merck, 95012613, RRID: CVCL_2164), and a healthy mouse osteoblast cell line, MC3T3-E1 (ATTC, CRL-2593, RRID: CVCL_0409), were included for further validation. Locally advanced prostate cancer cells, LNCaP (lymph node metastasis), were cultured in RPMI 1640 (PanBiotech, P04-18500) medium, supplemented with 1 % penicillin-streptomycin (PS) (Thermo, 15140122) and 10 % fetal bovine serum superior (FBS) (Merck, S0615). Healthy prostate cells, PNT2, were cultured in the same medium supplemented with 1 % PS, 10 % FBS, and 1 % 200 mM L-glutamine (Thermo, A2916801). For osseous metastasized prostate cancer, PC3 (bone metastasis) and VCaP (bone marrow metastasis) cells were cultured in DMEM F12 (PanBiotech, P0-41500) and DMEM (PanBiotech, P04-03550), respectively, both supplemented with 1 % PS and 10 % FBS. Additionally, MC3T3-E1 was cultured in αMEM Eagle (PanBiotech, P04-21250) medium, supplemented with 1 % PS and 10 % FBS to differentiate to mature osteoblast. All supplement amounts provided are given in (v/v). Cells were maintained in a humidified atmosphere at 37 °C and 5 % CO_2_. Medium was changed every 2 to 3 days and cells were sub-cultured at 80 % cell density by incubation with 0.25 % trypsin-ethylenediaminetetraacetic acid (tEDTA) solution (Thermo, 25200056) at 37 °C for 2 to 10 min. Consecutively, cells were centrifuged for 5 min at 100 × g at room temperature (RT). LNCaP (40,000 cells/cm^2^), VCaP (70,000 cells/cm^2^), PC3 (30,000 cells/cm^2^) as well as PNT2 (30,000 cells/cm^2^) and MC3T3-E1 (10,000 cells/cm^2^) were seeded on T-flasks for long term cultivation and on plastic or elastomer substrates for individual experiments. For cultivation of LNCaP and VCaP, T-flasks were coated with 0.01 % human fibronectin solution (FN) (Merck, FN0635) as described previously.^59^ FN-coating was used for all cell experiments to ensure high comparability. A hemocytometer was used for cell counting. PC3 and PNT2 cells were obtained from certified commercial suppliers (ATCC and Merck, respectively). MC3T3, LNCaP, and VCaP cells were characterized upon receipt using a validated cell line-specific mRNA marker panel (PCA3, AR, PSMA, and FR1α). Cell morphology was monitored daily, and molecular characterization was repeated at least every three months. All cell lines were routinely tested for mycoplasma contamination using a PCR-based assay (MycoSPY® PCR Kit, M020-50) every six months.

#### Human primary prostate cancer cell isolates

Prostates from male patients with histologically confirmed prostate cancer (Gleason scores 7b and 9) were used to isolate primary cells to assess their cultivability and cellular responses on physio-elastic PDMS- and control substrates (glass). Based on preoperative MRI-diagnostics, an experienced pathologist collected a 10-mm tissue core from respective tumor lesions. Primary cells derived from Gleason 7b specimen were isolated according to established protocols.^60^ In contrast, Gleason score 10 tumor tissue was processed using an enzymatic and mechanical dissociation approach to maximize cell yield and viability from highly aggressive, densely cellular samples. Tissue dissociation was performed using the gentleMACS™ Octo Dissociator (Miltenyi Biotec, 130-134-029) in combination with the Human Tumor Dissociation Kit (Milteny Biotec, 130-095-929), following the manufacturer’s instructions. Approximately 400-600 mg of each tumor core was processed. After isolation, cell suspensions were filtered, centrifuged for 7 min at 300 × g at RT and cell clusters smaller 70 μm were seeded onto FN-coated plastic and elastomer-coated 24-wells with an elasticity of 90 kPa (PDMS). Primary cells were cultivated in humidified atmosphere at 37 °C and 5 % CO_2_ using hESC StemPro medium (Thermo, A1000701) supplemented with 10 nM R1881 (Merck, R0908), 2 μM Y-27632 (Merck, Y0503-5MG), 10 μg/mL Gentamicin (v/v) (Thermo, 15710064) and 8 ng/mL human bFGF (v/v) (Thermo, PHG0264). Supernatants were collected every two days and centrifuged to retain loosely adherent cell clusters. Resulting pellets were gently resuspended in fresh medium and returned to corresponding wells.

#### Ethics approval

The use of patient samples within this study was approved by the local ethics committee of the medical faculty of the Rheinisch Westfälische Technische Hochschule (RWTH) Aachen according to § 6 Abs. 2 GDSG NRW and § 2 Abs. 1 GDSG NRW. Following patient consent for our project with the internal reference EK23-051 (6th February 2024), the PCa tissue was processed accordingly for the isolation of primary cells. Analyses using archived patient material were conducted taking the internal reference EK25-023 (23rd January 2025) into account. As this study focuses on prostate cancer, all models reflect male-derived, prostate-specific biology and tissue mechanics. The findings therefore cannot be directly generalized to other tumor entities or female patients. However, the underlying principle of incorporating physiologically relevant physical cues into cancer models may be broadly transferable and warrants validation in tissue- and tumor-specific models across sexes.

### Method details

#### Fabrication of flat elastomer substrates

To analyze the impact of tumor microenvironment (mTME) and metastatic tumor niche (MTN) elasticity (durotaxis) on cellular functionality and phenotype in 2D culture systems, physiologically relevant stiffness levels for prostate cancer were recapitulated. Elastic substrates were prepared to depict specific tissue-related elasticities as described previously.^10^ For this, Sylgard® 184 (PDMS) (VWR, DOWC25100445) was used and mixed in various base (A) to crosslinker (B) ratios (R) (R; A to B, (w/w)). Mixing ratios were calculated from existing calibrations and substrates were prepared as described previously.^61^ Briefly, after weighing and mixing for at least 2 min, the mixtures were degassed for 15 min at RT. After degassing, multi-well plates (Thermo, 142475, 167008) were coated with either 150 μL (24-well) or 50 μL (96-well) of different elastomer compositions. Following, the elastomer-coated plates were incubated on a 3D-shaker for 15 min at 5 rpm at RT. Cross-linking occurred at 50 °C overnight. After curing, respective elasticities were measured with nanoindentation. Effective Young’s modulus (E_eff_) of 1 kPa (R; 70 to 1 – bone marrow), 50 kPa (R; 40 to 1 – lymph node), 90 kPa (R; 35 to 1 – prostate cancer) and 1600 kPa (R; 10 to 1 – trabecular bone) were validated and used up to a deviation of 10 %. Sterilization of elastomer substrates was performed by washing once with 1 mL of 98 % propan-2-ol and 1 mL of 1x phosphate buffered saline (PBS). Afterwards, substrates were dried completely under sterile conditions for at least 1 h before use.

#### Fabrication of hydrogel patches

To investigate cell functionality under the influence of mTME- and MTN-specific elasticity (durotaxis) and defined topographies (topotaxis), customized hydrogel patches were fabricated using PEGDA (575 Da) (Merck, 455008) and GMA (Merck, 779342) crosslinked with lithium phenyl-2,4,6-trimethylbenzoylphosphinate (LAP) (Merck, 900889). LAP concentrations were maintained at 1 wt.% across all hydrogel formulations. Soft hydrogel patches were prepared with 15 wt.% PEGDA and 0.15 wt.% GMA (40 kPa structured (S)), while stiff hydrogel patches contained 20 wt.% PEGDA and 0.2 wt.% GMA (115 kPa S). Structured hydrogel patches were fabricated in microfluidic channels via stop-flow lithography (SFL) as described previously^25^ using a hexagonal arranged dot patterning mask (see Figures 6A–6C). Exposure parameters of the structured hydrogel patches were at 858 mW for 858 ms illumination time, resulting in an energy density of approximately 184 mJ/mm^2^. Then hydrogel patch types were flushed out of the channel as described previously.^25^ The selection of a hexagonal pattern was chosen to represent the large voids and dense areas known from histological surgery specimen (compare Figure S1) in one culture substrate at once. After fabrication, all hydrogel patch types were dispersed in 1x PBS, centrifuged for 5 s in a microcentrifuge, and the supernatant was removed by pipetting, repeating the process three times. For the cell culture preparation, all hydrogel patch types were transferred into 12-well cell culture slides (Ibidi GmbH, 81201) and exposed to UV light (365 nm, 5.2 mW/cm^2^, 15 min) for sterilization. Afterwards, hydrogel patches were washed three times with 200 μL of 1x PBS for 5 min. Until further use, all purified hydrogel patch types were stored at 4 °C in 1x PBS.

#### Elasticity testing

Mechanical properties of elastomer-coated substrate and hydrogel patches were determined using a nano indenter (Optics11Life, Pavone). A cantilever probe with a spherical tip (49.5 μm radius, 0.45 N/m stiffness) was installed. The cantilever probe was calibrated in deionized water, and all indentations were performed under the same conditions (RT, 1 μm/s indentation velocity, 7 μm indentation depth). Each hydrogel patch sample was prepared similarly as in the experiments and measured in deionized water at least three times at multiple positions. The E_eff_ was derived from the recorded indentation curves using the Hertzian contact model (R2 > 0.95) via the device-associated software (Optics11Life, V2.5).

#### Cell growth analysis

Prostate cancer cell lines (LNCaP, PC3 and VCaP) were seeded on plastic and elastomer-coated 24-well plates with an elasticity of 90 kPa (PDMS). Cells were cultivated for 48 h and then trypsinized and centrifuged as described above. Afterwards, supernatants were discarded, and cells were resuspended in 1 mL medium. Next, 200 μL of each cell suspension was diluted in 6 mL CASYton (OMNI Life Science, 5651808). Cell numbers were measured using the CASY Cell Counter & Analyzer (OMNI Life Science). Detached and dead cells were included in the measurement. The integrated CASY Software was used to determine the effect of elasticity on cell growth characteristics.

#### Lactate dehydrogenase assay

Prostate cancer cell lines were seeded on plastic as well as elastomer-coated 96-well plates with an elasticity of 90 kPa (PDMS) and were cultivated for 24 h to determine lactate dehydrogenase (LDH) activity. For quantification of spontaneous LDH activity the CyQuant™ LDH Cytotoxicity Assay Kit (Thermo, C20300) was used according to the manufacturer's instructions. Briefly, 10 μL of distilled water was added to each well. The plate was then incubated for 45 min at 37 °C. Afterwards, 50 μL of the supernatant was transferred to a new 96-well plate and mixed with 50 μL of Reaction Mixture, following incubation at RT for 30 min, protected from light. Then, 50 μL of Stop Solution was added and mixed by gentle tapping. Subsequently, absorbances were measured in a set of triplicates with a SpectraMax® iD3 Microplate Reader (Molecular Devices) and its integrated software at 490 nm as well as 680 nm (background). Finally, determination of spontaneous LDH activity was performed by subtracting background and negative control values (only cell culture medium) from cell samples. The resulting values for cells cultivated on elastomer-coated plates were normalized to the activity of cells cultured on plastic and plotted for each cell line.

#### Immunofluorescence staining

For the immunofluorescence analysis of cytoskeletal components paxillin and actin, prostate cancer cells were seeded onto elastomer coated 24-well plates with elasticities ranging from 1 kPa to 1600 kPa and hydrogel patches with elasticities in the range of 50 kPa and 90 kPa (see Figure S6). After cultivation for 48 h, fixation was performed with 4 % formaldehyde (Otto Fischar, 02653048) for 20 min at 37 °C. Subsequently, samples were incubated with 30 mM glycine (Merck, 67419) for 10 min at RT. Cell membranes were permeabilized with 0.1 % (hydrogel patches) or 2.5 % (elastomer substrates) Triton X-100 (v/v) (Merck, X100) for 2 min or 5 min. Cells were washed with 1x PBS for 5 min. Non-specific protein binding was blocked with 5 % (w/v) bovine serum albumin (BSA) (Merck, A-7906) diluted in 1x PBS (blocking buffer) for 1 h. Primary antibody, Paxillin Monoclonal Antibody (5H11, 1:100, (v/v)) (Thermo, #AHO0492, RRID: AB_2536312), was incubated in blocking buffer solution for 3 h. Samples were washed three times with blocking buffer solution. Secondary antibody, goat anti-mouse IgG (H+L) Alexa Fluor™ 568 nm (1:400, (v/v)) (Thermo, A-11004, RRID: AB_2534072), and Phalloidin Alexa Fluor™ 488 nm (1:1000, (v/v)) (Thermo, A12379, RRID: AB_2315147), were diluted in blocking buffer solution and added for 1 h. Samples were washed again with 1x PBS for 5 min. Nucleus staining was performed with Hoechst 33342 Ready Flow Reagent (Thermo, R37165), 1:20,000 (v/v) diluted in 1x PBS for 5 min, followed by three washing steps with 1x PBS. In addition to cell culture samples, 3 μm thick formalin-fixed paraffin-embedded (FFPE) tissue sections from pathologically confirmed prostate cancer and benign tissue were used for immunofluorescence. Furthermore, FFPE tissue sections derived from the primary tumor site of the patients with Gleason 7b and Gleason 10 were included. For all patient samples, a representative section was hematoxylin and eosin (H&E) stained and annotated by an experienced uro-oncological pathologist. Preparation of tissue sections, deparaffinization, antigen retrieval, non-specific antigen blocking and general staining procedure was performed as described previously.^59^ Actin and nucleus staining was carried out as described above for cell cultures. Primary antibodies for PSMA (NSJ Bioreagents, USA, F43183, LOT: 110622bw) and for androgen receptor (Merck, 06-680, RRID: AB_310214) were used and incubated overnight at 4 °C at a dilution of 1:500 (v/v) in blocking buffer. Secondary antibody, anti-rabbit Alexa Fluor™ 488 (Thermo, A-11008; 1:400, RRID: AB_143165) was used, and incubated for 1 h at RT. After washing with 1x PBS three times, sections were covered with Immuno-Mount (Epredia, 9990402) and a coverslip (Engelbrecht, K12460). For all immunofluorescence samples, staining and wash solutions were prepared in 1x PBS. Incubation steps were performed at RT. Hydrogel patches were always kept moisturized. Samples were stored at 4 °C until microscopy.

#### Quantification of chemosensitivity

The LIVE/DEAD™ Viability/Cytotoxicity Kit (Thermo, L3224) was used to characterize the viability of cells after treatment with docetaxel (Selleckchem, S1148) and Enzalutamide (ENZA) (Selleckchem, S1250) as established prostate cancer therapeutic agents. For this, prostate cancer cell lines were seeded on plastic and elastomer coated 24-well plates (LNCaP: 50 kPa, 90 kPa; PC3 and VCaP: 1 kPa, 1600 kPa; PDMS) for 24 h. Both agents were dissolved in 100 % dimethyl sulfoxide (DMSO) to create stock solutions of 10 μM (Docetaxel) and 10 mM (ENZA), respectively, and further diluted into the respective medium to achieve the desired final concentrations. All cells were treated with 10 nM docetaxel for 48 h. ENZA treatment was performed as described previously for 4 days (d) using concentrations of 0.1 – 10 μM.^62^ Following treatment cell viability was characterized according to the manufacturer’s staining protocol. Briefly, cells were washed with 500 μL of 1x PBS, detached by treatment with 0.25 % tEDTA-solution and centrifuged. Supernatants were discarded, and cells were carefully resuspended in 250 μL of their respective culture medium. Subsequently, 0.25 μL of the kit’s reactive dye was added and incubation was carried out for 30 min on ice, protected from light. Samples were washed twice with 500 μL of 1x PBS via centrifugation. For determination of cell viability, an untreated control and a positive dead control (treated with 500 μL of 70 % ethanol for 2 min at RT) were included. Quantification was performed either by flow cytometric analysis in 500 μL of 1x PBS or by absorbance measurements (for more method details see Flow Cytometer Analyses). Absorbance values were measured using the SpectraMax® iD3 Microplate Reader (Thermo).

#### PDMS-docetaxel interaction

To ensure accurate assessment of cell viability following docetaxel exposure, its concentrations in the media were analyzed using liquid chromatography-tandem mass spectrometry (LC-MS/MS). The LC-MS/MS system comprised an Agilent 1290 Infinity II UHPLC coupled with a SCIEX QTRAP6500+ triple quadrupole mass spectrometer. A solution of 100 nM docetaxel in 0.5 mL medium composed of DMEM F12 supplemented with 10 % FBS and 1 % PS was incubated on plastic and elastomer-coated 24-well plates of different elastomer compositions (1 kPa to 1600 kPa; PDMS) for 24 h at 37 °C. Following incubation, 40 μL of the medium supernatant was subjected to protein precipitation with 160 μL of methanol containing 0.1% (v/v) formic acid (VWR Chemicals, 84865.100). The mixture was vortexed thoroughly and centrifuged at 17,000 × g for 20 min at 4 °C. Subsequently, 100 μL of the resulting supernatant was transferred to LC vials for analysis. For LC-MS/MS, 10 μL of each sample was injected and separated using an Agilent Poroshell 120 EC-C18 column (1.9 μm, 2.1 × 50 mm; part number 699675-902, Agilent Technologies). The mobile phases consisted of 10 mM ammonium acetate with 0.1 % (v/v) formic acid in water (Phase A) and methanol (Phase B). The chromatographic method spanned 4 min at a flow rate of 0.6 mL/min, with the following gradient profile: 5 % B at 0 min, linearly increasing to 90 % B at 1.5 min, holding at 90 % B until 2.5 min, and returning to the initial 5 % B at 2.7 min, which was maintained until 4 min. Docetaxel quantification was performed using multiple reaction monitoring (MRM) in electrospray ionization positive mode, targeting the sodium adduct. The MRM transitions were as follows: 830.1 > 549.2 m/z (quantifier, collision energy (CE) 39 V), 830.1 > 304.1 m/z (qualifier 1, CE 35 V), and 830.1 > 248 m/z (qualifier 2, CE 45 V). A calibration curve was constructed using six non-zero standards with concentrations of 5, 10, 50, 100, 200, and 500 nM in the medium. A weighting factor of 1/x was applied, yielding a correlation coefficient (r^2^) of 0.98.

#### Physio-elastic Boyden Chamber

A modified Boyden Chamber experiment was used to investigate cell invasion (see Figure 4A). For this purpose, track-etched membranes filters (it4ip S.A, 1000M25/511M800/13) were coated by rinsing with 0.5 % solution of ethanolic acid and twice with distilled water, followed by boiling in 500 mg/L gelatin solution for 1 h and consecutive drying at 50 °C for 2 h. The bottoms of the Boyden Chambers were filled with 20 μL PDMS-mixture with elasticities of 1 kPa, 50 kPa, 90 kPa and 1600 kPa and were cured at 50 °C overnight (see Figure 4A: [3]). Sterilization with propan-2-ol and FN-coating was performed as described above. Then, PNT2 (50 kPa and 90 kPa) and differentiated MC3T3-E1 (1 kPa and 1600 kPa) as baiting cells were seeded onto the PDMS coated Boyden Chambers bottoms and cultivated for 48 h (see Figure 4A: [2]). Membrane filters and coverslips were then assembled to close the Boyden Chambers, and prostate cancer associated cell lines were added on top in FBS-free medium (see Figure 4A: [1]). After cultivation for 4 h, the membrane filters were removed and fixed with 4 % methanal for 10 min at RT, followed by Hoechst 33342 Ready Flow Reagent (1:20.000, (v/v), diluted in 1x PBS) staining for 5 min. Samples were stored at 4 °C until microscopy.

#### Quantification of angiogenic cytokines

The release of angiogenic cytokines of baiting cells in the modified Boyden Chamber experiment was investigated using the LEGENDplex™ Human Angiogenesis Panel 1 (Biolegend, 741215), which allows the simultaneous quantification of several key cytokines involved in the process of angiogenesis. This includes interleukin 6 (IL-6), angiopoietin-1 (Ang.1), angiopoietin-2 (Ang-2), epidermal growth factor (EGF), fibroblast growth factor (FGF), interleukin 8 (CXCL8 (IL-8)), platelet endothelial cell adhesion molecule (PECAM-1), placental growth factor (PlGF), vascular endothelial growth factor (VEGF) and tumor necrosis factor (TNF-α). For this purpose, PNT2 and MC3T3-E1 were seeded onto plastic and elastomer-coated 24-well plates with elasticities of 50 kPa and 90 kPa (PNT2) or 1 kPa and 1600 kPa (MC3T3-E1). Cells were cultured without media change. Supernatants were collected after 24 h, 48 h and 72 h through centrifugation to remove dead cells and stored at -80 °C until use. The assay was performed according to the supplier’s protocol for V-bottom plates. Briefly, supernatants were incubated with the kit’s capture beads overnight at 4 °C. Then, the kit’s detection-antibody solution was added and incubated for 1 h at RT. After washing the samples for 5 min at RT, flow cytometric analysis was performed in 150 μL of the kit’s 1x wash buffer. All incubation steps were performed on a 2D-shaker at 250 rpm. Cytokine concentration was calculated based on a standard curve using the LEGENDplex data analysis software (version 2023-02-15). Quantification of cytokines was repeated four times per sample.

#### Quantification of prostate cancer specific markers within primary cell cultures

Total RNA was isolated on day 10 using the RNeasy Midi Kit (Qiagen, 77144) and 15-20 ng were transcribed into cDNA with the Maxima First Strand cDNA Synthesis Kit (Thermo, K1641). All procedures were carried out according to established protocols as previously described.^59^ For qRT-PCR analyses, TaqMan® assays (Thermo Fisher Scientific) targeting Androgen Receptor (AR) (Hs00171172_m1), Prostate Cancer Antigen 3 (*PCA3*) (Hs01371939_g1), and Prostate-Specific Membrane Antigen (FOLH1) (Thermo, Hs00379515_m1) were employed. Glyceraldehyde 3-phosphate dehydrogenase (GAPDH) (Hs02786624_g1) was used as endogenous reference control. cDNA amplification was carried out with a StepOnePlus™ Real-Time PCR System (Thermo, 4376600) using the instrument’s standard cycling program. Ct values were quantified with the StepOne™ Software (v2.3), and relative expressions were calculated using ΔΔCt-values normalized to GAPDH. Then, marker expression was calculated relative to the plastic control for each Gleason score.

#### Flow cytometer analysis

Quantification of the release of angiogenic cytokines and cell viability staining was performed using the FACSCanto™ Clinical Flow Cytometry System II (BD Biosciences). Cell populations were gated separately by cell granularity and size (forward and side scatter signals). The integrated 3-laser 8-colour (2-4-2) laser module with different excitation wavelengths was used to quantify angiogenic cytokine expression (575 nm) and cell viability (633 nm). At least 5,000 (cytokine expression) or 10,000 (cell viability) events were analyzed using appropriate flow cytometer instrument settings. Flow cytometer analysis was supported by the Flow Cytometry Facility, a core facility of the Interdisciplinary Center for Clinical Research (IZKF) Aachen within the Faculty of Medicine at RWTH Aachen University.

#### Co-culture-elasticity migration assay (CEMA)

Additional Co-Culture Elasticity Migration Assays (CEMA) were performed to investigate substrate-dependent stromal signaling and its impact on tumor cell migration. Non- and elastomer-coated 24-well plates (50 kPa and 90 kPa, PDMS) were prepared, and Culture-Inserts were applied as described for MEMA. LNCaP and PNT2 cells were seeded into the designated chambers, and, after complete cell attachment, the wells were filled with media. Cells were cultured for 48 h to allow cytokine-mediated cell-cell interactions without medium exchange. Subsequently, Culture-Inserts were removed and migration of LNCaP cells was analyzed. Corresponding monoculture controls were included to determine baseline migration for each substrate condition.

#### Multi-Elasticity Migration Assay (MEMA)

A self-developed Multi-Elasticity Migration Assay (MEMA, see Figures 3A–3C) was used to analyze the migration rate of prostate cancer associated cell lines at different elasticities between 1 kPa to 1600 kPa within one sample. To prepare the MEMA, a customized silicone stamp was inserted into a 12-well plate (*Thermo, 150628*). The holes of the stamp (see Figure 3B: SE1 [1], SE2 [2]) were then filled with 100 μL of PDMS mixture with elasticities of either 1 kPa or 50 kPa (SE1) and 90 kPa (SE2). Subsequently, the plate was cured at 50 °C for 1 h. After curing, the silicone stamp was carefully removed and 20 μL of a PDMS mixture with an elasticity of 1600 kPa (see Figure 4C: SE3 [3]) was pipetted into the center of partially cross-linked elastomers, allowing the creation of a multi-elasticity zone. Subsequently, complete crosslinking was performed at 50 °C overnight. Sterilization and FN-coating of the MEMA was performed as described above. Afterwards, FN solution was removed, and the MEMA was dried for 5 min at RT. Next, a Culture-Insert (Ibidi, 81176) was inserted onto the SE3 of the MEMA and dried again for 5 min at RT. Following, prostate cancer cells were seeded into their designated areas of the Culture-Insert (see Figure 3) and cells were cultivated for 48 h until a confluent cell layer has been expanded. Then, Culture-Insert was removed, and cell migration was observed by live cell microscopy (Zeiss Axio Observer). Here, the migrating front of the before expanded confluent cell layer was evaluated towards the direction of the three different zones (SE1, leftwards; SE2, rightwards; SE3, up-/downwards) The construction and fabrication of the silicone stamp for preparation of MEMA was supported by the Scientific Workshop of the University Hospital RWTH Aachen.

#### Microscopy

Microscopy analysis was performed on a Zeiss Axio Observer microscope equipped with a fluorescence Colibri5 light source, a second LED light source, an Axiocam 503 Mono, an Apotome 3 and the corresponding Zen 3.8 software (RRID:SCR_013672). For each experiment, the microscope settings were adapted according to the required experimental setup and kept constant. To analyze the migration rates, following settings, MEMA = time series over 20 h with an imaging interval of 2 h; hydrogel patches = time series of 6 h with an imaging interval of 10 min, were used. Live cell imaging was performed under cell culturing conditions using the Zeiss incubation unit. Imaging was conducted using an EC-Plan-Neofluar 10x/0.30 Ph1 (cell growth analysis; migration analysis; cell invasion; morphology analysis; cytoskeleton analysis) and a Plan Apochromat 40x/1.4 oil objective (high resolution imaging of immunofluorescence samples). Reconstruction of apotome images was carried out using Zen 3.8 software, which integrated optical sectioning, local bleaching and phase correction. Immunofluorescence imaging of tissue sections was performed within H&E-annotated tumor regions. A Leica DM IL LED microscope (Leica Microsystems, Wetzlar, Germany) was used for microscopic analyses of isolated primary cells with an HI PLAN CY 10×/0.25 objective to visualize cell proliferation and morphology over 10 d.

### Quantification and statistical analysis

#### Image analysis

All images were analyzed using ImageJ (National Institutes of Health, RRID:SCR_003070). For analyzing cell morphology on elastomer substrates and hydrogel patches, single cells were marked using the “polygon selection” tool. Perimeter, diameter, area and roundness of selected cells were analyzed. A roundness value of 1 was considered as an ideal circular cell. In addition, the orientation of actin filaments was determined using the FibrilTool plugin (RRID:SCR_016773)^63^ in all marked cells. The overall total intensities of actin, paxillin, PSMA and androgen receptor were quantified using the “measure” feature. For invasion analysis in Boyden Chamber membrane filters, cell nuclei were manually selected and counted via the “measure” feature. For quantification of migration speed with MEMAs and on hydrogel patches, images were merged into a stack. Then, 10 different equidistantly distributed start and end points were analyzed. Values were generated and summarized from 12 different positions of three independent samples.

#### Statistical and graphical analysis

Calculations were performed in Microsoft Excel (MS Office 2019, RRID:SCR_016137) using the Real Statistics Resource Pack software (Release 9.6.1). Analysis of normal distribution and homogeneity of variance was carried out utilizing the Kolmogorov-Smirnov test and Levene's test, respectively. All data are given as means including standard error of the mean (mean +/- SEM). The use of standard error of the mean was chosen as it quantifies the degree of uncertainty associated with the mean estimation process, providing a relevant indicator of the statistical confidence in the reported group means. Each data set was based on at least three independent experiments unless otherwise stated. Calculated data were generated from each experiment and combined accordingly. The non-parametric Mann-Whitney U (MWU) test was used to determine statistical differences between samples, accounting for data not meeting the assumptions of normality and homogeneity of variances. The null hypothesis stated that there were no differences among group ranks. A *p*-value of ≤ 0.05 was considered as significant. Results with significances of ≤ 0.05, ≤ 0.01 and ≤ 0.001 were marked with one to three corresponding symbols. Figures and graphs were created using Microsoft PowerPoint (MS Office 2019) and Origin 2019 64Bit (OriginLab Graphing & Analysis). Technical drawings were created using Inventor Professional 2023. Renderings were created either in Inventor Professional 2023 or Blender 3.1.

## References

[1] Rofstad E.K., Halsør E.F. (2002). Hypoxia-associated spontaneous pulmonary metastasis in human melanoma xenografts: involvement of microvascular hot spots induced in hypoxic foci by interleukin 8. Br. J. Cancer.

[2] Li Z., Bao S., Wu Q., Wang H., Eyler C., Sathornsumetee S., Shi Q., Cao Y., Lathia J., McLendon R.E. (2009). Hypoxia-Inducible Factors Regulate Tumorigenic Capacity of Glioma Stem Cells. Cancer Cell.

[3] Gerlinger M., Rowan A.J., Horswell S., Math M., Larkin J., Endesfelder D., Gronroos E., Martinez P., Matthews N., Stewart A. (2012). Intratumor Heterogeneity and Branched Evolution Revealed by Multiregion Sequencing. N. Engl. J. Med..

[4] Miki J., Rhim J.S. (2008). Prostate cell cultures as in vitro models for the study of normal stem cells and cancer stem cells. Prostate Cancer Prostatic Dis..

[5] Peehl D.M. (2004). Are primary cultures realistic models of prostate cancer?. J. Cell. Biochem..

[6] Niranjan B., Lawrence M.G., Papargiris M.M., Randell S.H., Fulcher M.L. (2012). *Epithelial Cell Culture Protocols*. Vol 945. Methods in Molecular Biology.

[7] Sung H., Ferlay J., Siegel R.L., Laversanne M., Soerjomataram I., Jemal A., Bray F. (2021). Global Cancer Statistics 2020: GLOBOCAN Estimates of Incidence and Mortality Worldwide for 36 Cancers in 185 Countries. CA Cancer J. Clin..

[8] Ferlay J., Colombet M., Soerjomataram I., Parkin D.M., Piñeros M., Znaor A., Bray F. (2021). Cancer statistics for the year 2020: An overview. Int. J. Cancer.

[9] Kanagaraju V., Ashlyin P.V.K., Elango N., Devanand B. (2020). Role of transrectal ultrasound elastography in the diagnosis of prostate carcinoma. J. Med. Ultrasound.

[10] Ji Y., Ruan L., Ren W., Dun G., Liu J., Zhang Y., Wan Q. (2009). Stiffness of prostate gland measured by transrectal real-time shear wave elastography for detection of prostate cancer: a feasibility study. Br. J. Radiol..

[11] Good D.W., Stewart G.D., Hammer S., Scanlan P., Shu W., Phipps S., Reuben R., McNeill A.S. (2014). Elasticity as a biomarker for prostate cancer: a systematic review. BJU Int..

[12] Yi B., Xu Q., Liu W. (2022). An overview of substrate stiffness guided cellular response and its applications in tissue regeneration. Bioact. Mater..

[13] Mierke C.T. (2024). Extracellular Matrix Cues Regulate Mechanosensing and Mechanotransduction of Cancer Cells. Cells.

[14] Mielnicka A., Kołodziej T., Dziob D., Lasota S., Sroka J., Rajfur Z. (2023). Impact of elastic substrate on the dynamic heterogeneity of WC256 Walker carcinosarcoma cells. Sci. Rep..

[15] Fan Y., Sun Q., Li X., Feng J., Ao Z., Li X., Wang J. (2021). Substrate Stiffness Modulates the Growth, Phenotype, and Chemoresistance of Ovarian Cancer Cells. Front. Cell Dev. Biol..

[16] Emon B., Song Y.J., Joy M.S.H., Kovour M.V., Prasanth K.V., Saif M.T.A. (2023). Mechanosensitive changes in the expression of genes in colorectal cancer-associated fibroblasts. Sci. Data.

[17] Jeon H., Tsui J.H., Jang S.I., Lee J.H., Park S., Mun K., Boo Y.C., Kim D.H. (2015). Combined Effects of Substrate Topography and Stiffness on Endothelial Cytokine and Chemokine Secretion. ACS Appl. Mater. Interfaces.

[18] Yui A., Oudin M.J. (2024). The Rigidity Connection: Matrix Stiffness and Its Impact on Cancer Progression. Cancer Res..

[19] Bertolio R., Napoletano F., Del Sal G. (2023). Dynamic links between mechanical forces and metabolism shape the tumor milieu. Curr. Opin. Cell Biol..

[20] Fernie A.R., Zhang Y., Sampathkumar A. (2020). Cytoskeleton Architecture Regulates Glycolysis Coupling Cellular Metabolism to Mechanical Cues. Trends Biochem. Sci..

[21] Lo W.C., Hsu W.L., Wang C.T., Cheng P.W., Liao L.J. (2019). Incorporation of shear wave elastography into a prediction model in the assessment of cervical lymph nodes. PLoS One.

[22] Handorf A.M., Zhou Y., Halanski M.A., Li W.J. (2015). Tissue Stiffness Dictates Development, Homeostasis, and Disease Progression. Organogenesis.

[23] Morgan E.F., Unnikrisnan G.U., Hussein A.I. (2018). Bone Mechanical Properties in Healthy and Diseased States. Annu. Rev. Biomed. Eng..

[24] Palchesko R.N., Zhang L., Sun Y., Feinberg A.W. (2012). Development of Polydimethylsiloxane Substrates with Tunable Elastic Modulus to Study Cell Mechanobiology in Muscle and Nerve. PLoS One.

[25] Steinbeck L., Paul R., Litke J., Karkoszka I., Wiese G.P., Linkhorst J., De Laporte L., Wessling M. (2025). Hierarchically Structured and Tunable Hydrogel Patches: Design, Characterization, and Application. Small.

[26] Moya L., Walpole C., Rae F., Srinivasan S., Seim I., Lai J., Nicol D., Williams E.D., Clements J.A., Batra J. (2023). Characterisation of cell lines derived from prostate cancer patients with localised disease. Prostate Cancer Prostatic Dis..

[27] Brady L., Gil Da Costa R.M., Coleman I.M., Matson C.K., Risk M.C., Coleman R.T., Nelson P.S. (2020). A comparison of prostate cancer cell transcriptomes in 2D monoculture vs 3D xenografts identify consistent gene expression alterations associated with tumor microenvironments. Prostate.

[28] El Harane S., Zidi B., El Harane N., Krause K.H., Matthes T., Preynat-Seauve O. (2023). Cancer Spheroids and Organoids as Novel Tools for Research and Therapy: State of the Art and Challenges to Guide Precision Medicine. Cells.

[29] Jouberton E., Voissiere A., Penault-Llorca F., Cachin F., Miot-Noirault E. (2022). Multicellular tumor spheroids of LNCaP-Luc prostate cancer cells as in vitro screening models for cytotoxic drugs. Am. J. Cancer Res..

[30] Rodríguez-Dorantes M., Cruz-Hernandez C.D., Cortés-Ramírez S.A., Robles-Flores M. (2021). *Cancer Cell Signaling*. Vol 2174. Methods in Molecular Biology.

[31] Valta M.P., Zhao H., Saar M., Tuomela J., Nolley R., Linxweiler J., Sandholm J., Lehtimäki J., Härkönen P., Coleman I. (2016). Spheroid culture of LuCaP 136 patient-derived xenograft enables versatile preclinical models of prostate cancer. Clin. Exp. Metastasis.

[32] Nguyen H.M., Vessella R.L., Morrissey C., Brown L.G., Coleman I.M., Higano C.S., Mostaghel E.A., Zhang X., True L.D., Lam H.M. (2017). LuCaP Prostate Cancer Patient-Derived Xenografts Reflect the Molecular Heterogeneity of Advanced Disease an--d Serve as Models for Evaluating Cancer Therapeutics: Prostate Cancer PDXs: LuCaP Series. Prostate.

[33] Saar M., Zhao H., Nolley R., Young S.R., Coleman I., Nelson P.S., Vessella R.L., Peehl D.M. (2014). Spheroid culture of LuCaP 147 as an authentic preclinical model of prostate cancer subtype with SPOP mutation and hypermutator phenotype. Cancer Lett..

[34] Liu Q.P., Luo Q., Deng B., Ju Y., Song G.B. (2020). Stiffer Matrix Accelerates Migration of Hepatocellular Carcinoma Cells through Enhanced Aerobic Glycolysis Via the MAPK-YAP Signaling. Cancers.

[35] Sartor O., Coleman R.E., Nilsson S., Heinrich D., Helle S.I., O'Sullivan J.M., Vogelzang N.J., Bruland Ø., Kobina S., Wilhelm S. (2017). An exploratory analysis of alkaline phosphatase, lactate dehydrogenase, and prostate-specific antigen dynamics in the phase 3 ALSYMPCA trial with radium-223. Ann. Oncol..

[36] Sharma D., Singh M., Rani R. (2022). Role of LDH in tumor glycolysis: Regulation of LDHA by small molecules for cancer therapeutics. Semin. Cancer Biol..

[37] Cascardo F., Anselmino N., Páez A., Labanca E., Sanchis P., Antico-Arciuch V., Navone N., Gueron G., Vázquez E., Cotignola J. (2021). HO-1 Modulates Aerobic Glycolysis through LDH in Prostate Cancer Cells. Antioxidants.

[38] Jiang B. (2017). Aerobic glycolysis and high level of lactate in cancer metabolism and microenvironment. Genes Dis..

[39] Crozet F., Levayer R. (2023). Emerging roles and mechanisms of ERK pathway mechanosensing. Cell. Mol. Life Sci..

[40] Mohamed A.A., Tan S.H., Xavier C.P., Katta S., Huang W., Ravindranath L., Jamal M., Li H., Srivastava M., Srivatsan E.S. (2017). Synergistic Activity with NOTCH Inhibition and Androgen Ablation in ERG-Positive Prostate Cancer Cells. Mol. Cancer Res..

[41] Gao J., Rong Y., Huang Y., Shi P., Wang X., Meng X., Dong J., Wu C. (2019). Cirrhotic stiffness affects the migration of hepatocellular carcinoma cells and induces sorafenib resistance through YAP. J. Cell. Physiol..

[42] Merino-Casallo F., Gomez-Benito M.J., Hervas-Raluy S., Garcia-Aznar J.M. (2022). Unravelling cell migration: defining movement from the cell surface. Cell Adhes. Migrat..

[43] Monteiro N.O., Fangueiro J.F., Reis R.L., Neves N.M. (2023). Replication of natural surface topographies to generate advanced cell culture substrates. Bioact. Mater..

[44] Buonvino S., Di Giuseppe D., Filippi J., Martinelli E., Seliktar D., Melino S. (2024). 3D Cell Migration Chip (3DCM-Chip): A New Tool toward the Modeling of 3D Cellular Complex Systems. Adv. Healthcare Mater..

[45] Geiger M., Marsico P., Pensold D., Wessling M., Zimmer-Bensch G., Linkhorst J. (2024). A Microfluidic Perfusion Culture Setup to Investigate Cell Migration in 3D Constrictions. Adv. Mater. Technol..

[46] Schwartz A.D., Barney L.E., Jansen L.E., Nguyen T.V., Hall C.L., Meyer A.S., Peyton S.R. (2017). A biomaterial screening approach reveals microenvironmental mechanisms of drug resistance. Integr. Biol..

[47] Liu C., Liu Y., Xie H.G., Zhao S., Xu X.X., Fan L.X., Guo X., Lu T., Sun G.W., Ma X.J. (2015). Role of three-dimensional matrix stiffness in regulating the chemoresistance of hepatocellular carcinoma cells. Biotechnol. Appl. Biochem..

[48] De La Cruz-López K.G., Castro-Muñoz L.J., Reyes-Hernández D.O., García-Carrancá A., Manzo-Merino J. (2019). Lactate in the Regulation of Tumor Microenvironment and Therapeutic Approaches. Front. Oncol..

[49] Rice A.J., Cortes E., Lachowski D., Cheung B.C.H., Karim S.A., Morton J.P., Del Río Hernández A. (2017). Matrix stiffness induces epithelial–mesenchymal transition and promotes chemoresistance in pancreatic cancer cells. Oncogenesis.

[50] Lei K., Kurum A., Kaynak M., Bonati L., Han Y., Cencen V., Gao M., Xie Y.Q., Guo Y., Hannebelle M.T.M. (2021). Cancer-cell stiffening via cholesterol depletion enhances adoptive T-cell immunotherapy. Nat. Biomed. Eng..

[51] Adekoya T.O., Richardson R.M. (2020). Cytokines and Chemokines as Mediators of Prostate Cancer Metastasis. IJMS.

[52] Mughees M., Kaushal J.B., Sharma G., Wajid S., Batra S.K., Siddiqui J.A. (2022). Chemokines and cytokines: Axis and allies in prostate cancer pathogenesis. Semin. Cancer Biol..

[53] Parmar D., Apte M. (2021). Angiopoietin inhibitors: A review on targeting tumor angiogenesis. Eur. J. Pharmacol..

[54] Johnson C.S., Cook L.M. (2023). Osteoid cell-derived chemokines drive bone-metastatic prostate cancer. Front. Oncol..

[55] Hoshino A., Costa-Silva B., Shen T.L., Rodrigues G., Hashimoto A., Tesic Mark M., Molina H., Kohsaka S., Di Giannatale A., Ceder S. (2015). Tumour exosome integrins determine organotropic metastasis. Nature.

[56] Chen F., Han Y., Kang Y. (2021). Bone marrow niches in the regulation of bone metastasis. Br. J. Cancer.

[57] Caspar A., Mostertz J., Leymann M., Ziegler P., Evert K., Evert M., Zimmermann U., Brandenburg L.O., Burchardt M., Stope M.B. (2016). In Vitro Cultivation of Primary Prostate Cancer Cells Alters the Molecular Biomarker Pattern. In Vivo.

[58] Buskin A., Scott E., Nelson R., Gaughan L., Robson C.N., Heer R., Hepburn A.C. (2023). Engineering prostate cancer in vitro: what does it take?. Oncogene.

[59] Hoffmann M., Ermler T.F., Hoffmann F., Alexa R., Kranz J., Steinke N., Leypold S., Gaisa N.T., Saar M. (2024). Therapeutic and Diagnostic Potential of Folic Acid Receptors and Glycosylphosphatidylinositol (GPI) Transamidase in Prostate Cancer. Cancers.

[60] Young S.R., Saar M., Santos J., Nguyen H.M., Vessella R.L., Peehl D.M. (2013). Establishment and serial passage of cell cultures derived from LuCaP xenografts. Prostate.

[61] Cesa C.M., Kirchgeßner N., Mayer D., Schwarz U.S., Hoffmann B., Merkel R. (2007). Micropatterned silicone elastomer substrates for high resolution analysis of cellular force patterns. Rev. Sci. Instrum..

[62] Devaux F., Beltagi K., Ermler T.F., Gerlach S., Herrmann A., Kranz J., Alexa R., Morgenroth A., Vogg A.T.J., Schäfer L. (2025). Androgen receptor blockade and its effect on PSMA-localization in prostate cancer: Implications for radioligand therapy. Biomed. Pharmacother..

[63] Boudaoud A., Burian A., Borowska-Wykręt D., Uyttewaal M., Wrzalik R., Kwiatkowska D., Hamant O. (2014). FibrilTool, an ImageJ plug-in to quantify fibrillar structures in raw microscopy images. Nat. Protoc..

