# Supplementary material for: Physiological elasticity and topography modulate in vitro prostate cancer cell behavior
Source: iScience. 2026 Sep 30;29(10):117623. doi: 10.1016/j.isci.2026.117623 (PMC13657116; doi:10.1016/j.isci.2026.117623)
Supplement: Supplementary file 1 [file mmc1.pdf]

iScience, Volume *29*

Supplemental information

Physiological elasticity and topography modulate *in vitro* prostate cancer cell behavior

Marco Hoffmann, Richard Paul, Anna Herrmann, Sven Gerlach, Paul Bacmeister, Thomas Frank Ermler, Marie Book, Radu Alexa, Axel Honné, Julian Peter Müller, Julia Kristin Brach, Petra Fischer, Jennifer Kranz, Matthias Wessling, and Matthias Saar

Supplementary Figures and Tables

Figure S1: Morphology and actin organization of primary cells across surgical tissue specimens.

Figure S2: Enhanced migratory behavior of LNCaP cells under physiologically relevant elastic culture conditions and in co-culture with PNT-2 cells.

Figure S3: Drug-substrate interactions under conventional plastic and physio-elastic culture conditions and therapy sensitivity of prostate cancer cells.

Figure S4: Primary prostate cancer cell isolation cultured on PDMS (90 kPa) and control substrates.

Figure S5: Quantification of relevant prostate cancer markers in primary prostate cancer tissue and cells cultured on PDMS (90 kPa) and control substrates.

Figure S6: Parameters of the created hydrogel patches.

Table S1: Cytokine expression of PNT-2 cells under conventional plastic and physio-elastic culture conditions.

Table S2: Cytokine expression of MC3T3-E1 cells under conventional plastic and physio-elastic culture conditions.

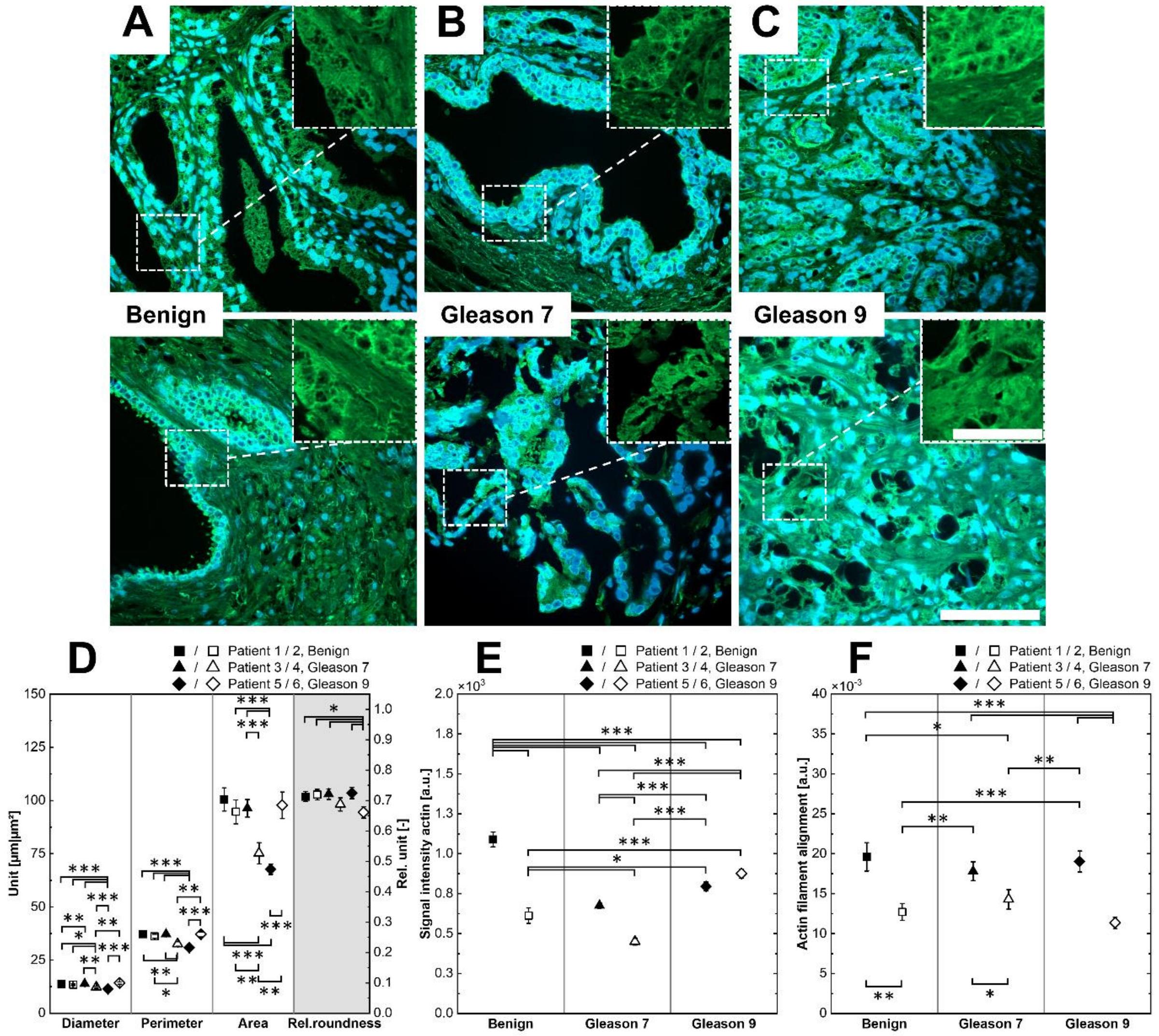

Figure S1: Morphology and actin organization of primary cells across surgical tissue specimens. *Cytoskeleton expression visualized by immunofluorescence staining for actin (green) and cell nucleus (blue) in benign tissue (**A**: Patient 1 ■/ Patient 2□), Gleason Score 7 (**B**: Patient 3 ▲/Patient 4△), and Gleason Score 9 (**C**: Patient 5 ◆/ Patient 6 ◇) surgical tissue specimens. (**D**) Qualitative evaluation of cell morphology. (**E**) Signal intensities of actin in cancer cells of prostate cancer tissue across the three health states in glandular areas. (**F**) Actin filament alignment quantified for the same cells. Data are represented as mean +/- SEM. Asterisks denote significance levels determined by Mann-Whitney U test (MWU): p ≤ 0.05 (*), p ≤ 0.01 (**), and p ≤ 0.001 (***), based on at least 60 cell analyses. Scale bar: 100 µm, Insert scale bar: 50 µm.*

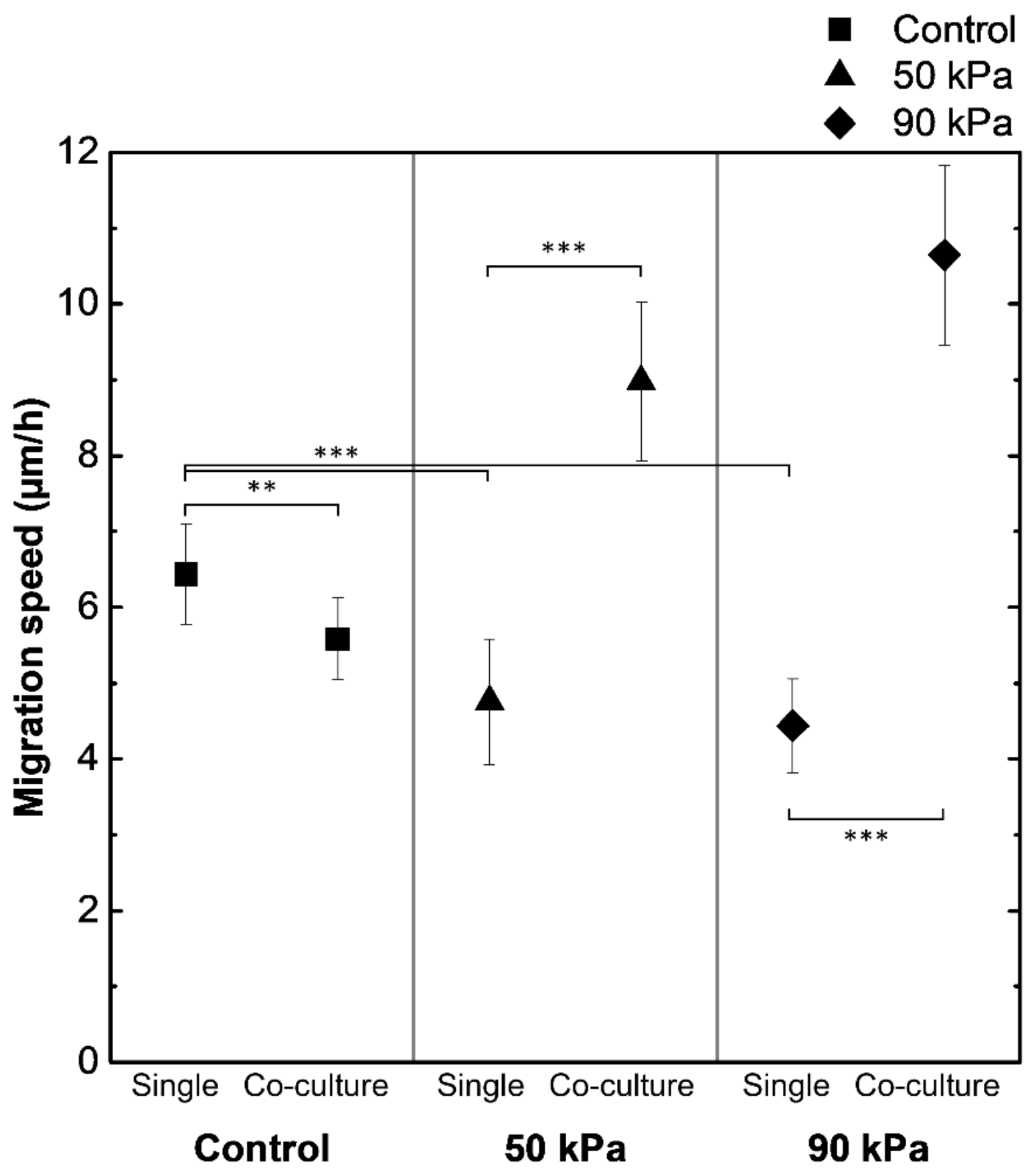

Figure S2: Enhanced migratory behavior of LNCaP cells under physiologically relevant elastic culture conditions and in co-culture with PNT-2 cells. *Migration speed (µm/h) of LNCaP cells was quantified in monoculture and in co-culture with PNT-2 cells under conventional plastic culture conditions (control) and physiologically relevant elastic substrate conditions (50 kPa and 90 kPa). Data demonstrate increased migratory activity under physio-elastic culture conditions, which was further enhanced in the presence of PNT-2 cells. Data are represented as mean +/- SEM. Asterisks denote significance levels determined by Mann-Whitney U test (MWU): $p \leq 0.05$ (*), $p \leq 0.01$ (**), and $p \leq 0.001$ (***), based on at least three biological replicates.*

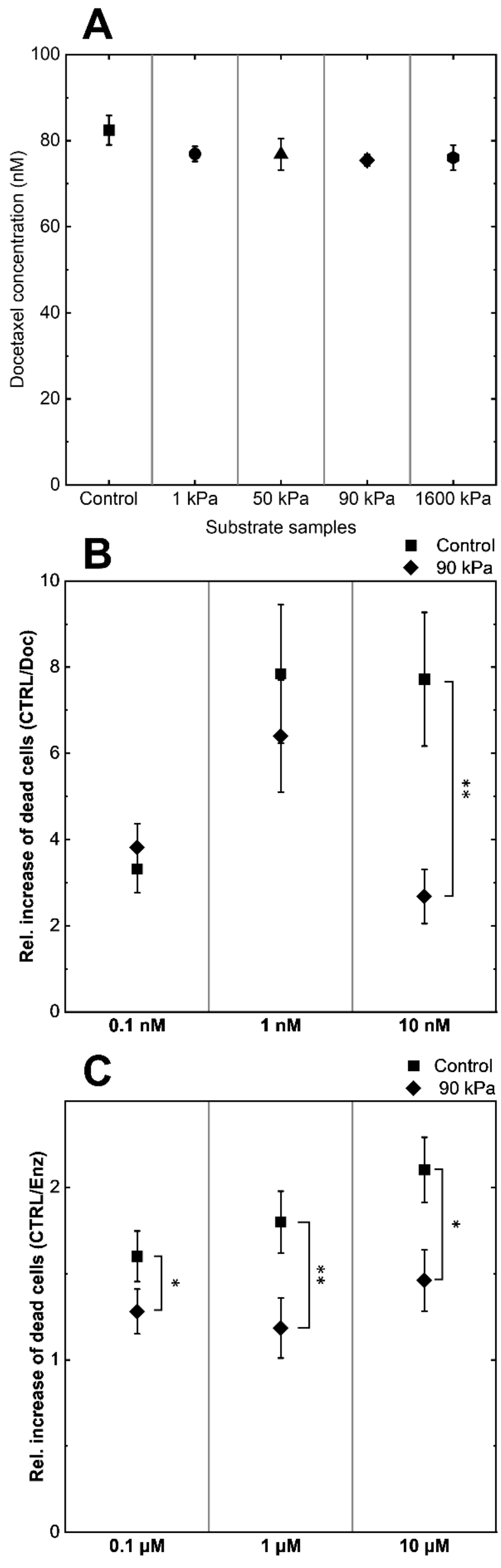

Figure S3: Drug-substrate interactions under conventional plastic and physio-elastic culture conditions and therapy sensitivity of prostate cancer cells. *Plastic control (■), and PDMS-substrates with elasticities of 1 kPa (●), 50 kPa (▲), 90 kPa (⬟) and 1600 kPa (◆) were incubated with 100 nM Docetaxel at 37 °C for 24 h (**A**). No statistically significant differences in the effective, medium-available concentrations of docetaxel were observed between experimental groups, as determined by one-way ANOVA followed by Dunnett's post hoc test. Each condition was tested in four replicates and a duplicate measurement. Quantification was conducted via ultra-high-performance liquid chromatography (UHPLC) coupled to a SCIEX QTRAP 6500+ triple quadrupole mass spectrometer. Drug sensitivity of LNCaP cells was assessed under conventional plastic and physio-elastic culture conditions. Cells were treated with docetaxel (0.1, 1, and 10 µM) (**B**) or the androgen receptor inhibitor enzalutamide (0.1, 1, and 10 nM) (**C**), and viability was quantified relative to untreated controls (ctrl) for each culture condition. Data are represented as mean +/- SEM. Asterisks denote significance levels determined by Mann-Whitney U test (MWU): p ≤ 0.05 (*), p ≤ 0.01 (**), and p ≤ 0.001 (***), based on at least three biological replicates.*

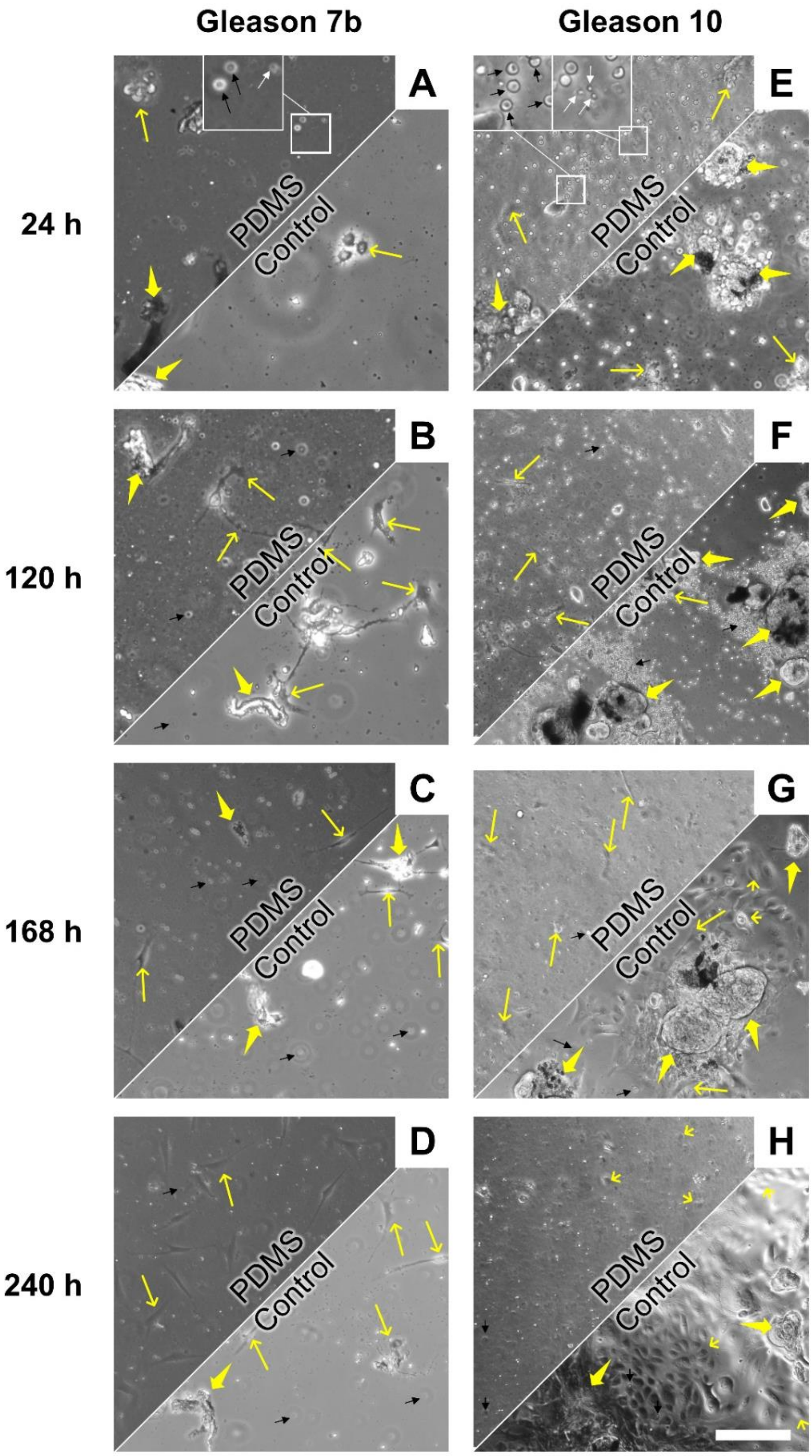

Figure S4: Primary prostate cancer cell isolation cultured on PDMS (90 kPa) and control substrates. *Tissue cores with histologically confirmed prostate cancer (Gleason scores 7b and 10) were dissociated either by hand together with collagenase (Gleason 7b) or with the help of a gentleMACS™ Octo Dissociator and the human tumor dissociation kit (Gleason 10) due to differences in tumor tissue composition and architecture. Pictures were taken after cultivation of 24 h (**A**, **E**), 120 h (**B**, **F**), 168 h (**C**, **G**) and 240 h (**D**, **H**) on PDMS (top left part of the combined images) and control substrates (bottom right part of the combined images). Due to media change, compounds like erythrocytes (black arrow, exemplary A, E inset), debris (white arrow) and non-adherent matrix or cell clusters (yellow pointy arrow) were partly lost. Yellow, roundish arrows index phenotypical stretching cells, whilst yellow short arrows index phenotypical roundish cells. Images were taken on arbitrary locations at the same magnification. Scale bar: 200 µm.*

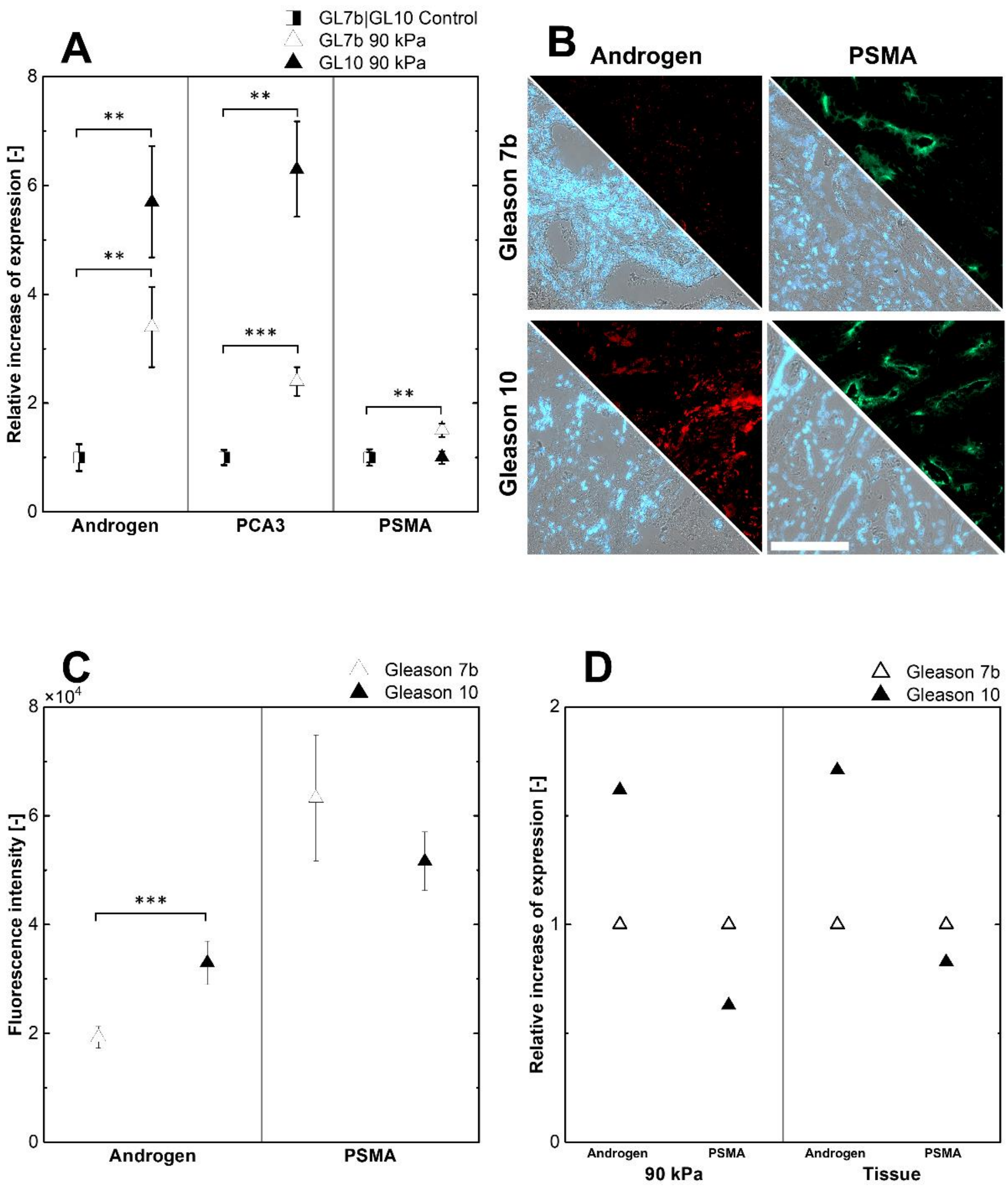

Figure S5: Quantification of relevant prostate cancer markers in primary prostate cancer tissue and cells on PDMS (90 kPa) and control substrates. *Total RNA amount was isolated on day 10 (240h) and analyzed using quantitative real time polymerase chain reaction method (qRT-PCR). Primary isolated cells showed significantly higher expression for androgen receptor (Androgen) and prostate cancer antigen (PCA3) on 90 kPa (PDMS; Gleason 7b (△ GL7b) / Gleason 10 (▲ GL10) compared with the glass control (◨ GL7b/GL10). For the prostate-specific membrane antigen (PSMA) a significant increase was observed for primary cells on 90 kPa only for Gleason 7b (**A**). Patient-derived tissue samples (Gleason 7b and Gleason 10) were stained using identical antibodies against androgen receptor (AR) and prostate-specific membrane antigen (PSMA) to characterize the receptor status of the originating patient material (**B**). Quantitative analysis of fluorescence intensities revealed higher AR expression in Gleason 10 patient samples, whereas higher PSMA expression was observed in Gleason 7b tissue (**C**). Importantly, these relative expression patterns of the relevant receptors were faithfully recapitulated in physiologically elastic culture conditions using primary tumor cells, demonstrating preservation of patient-specific molecular characteristics in vitro (**D**). Data are represented as mean +/- SEM. Asterisks indicate significance levels determined by Mann-Whitney U test (MWU): $p \leq 0.05$ (*), $p \leq 0.01$ (**), and $p \leq 0.001$ (***), based on one experiment.*

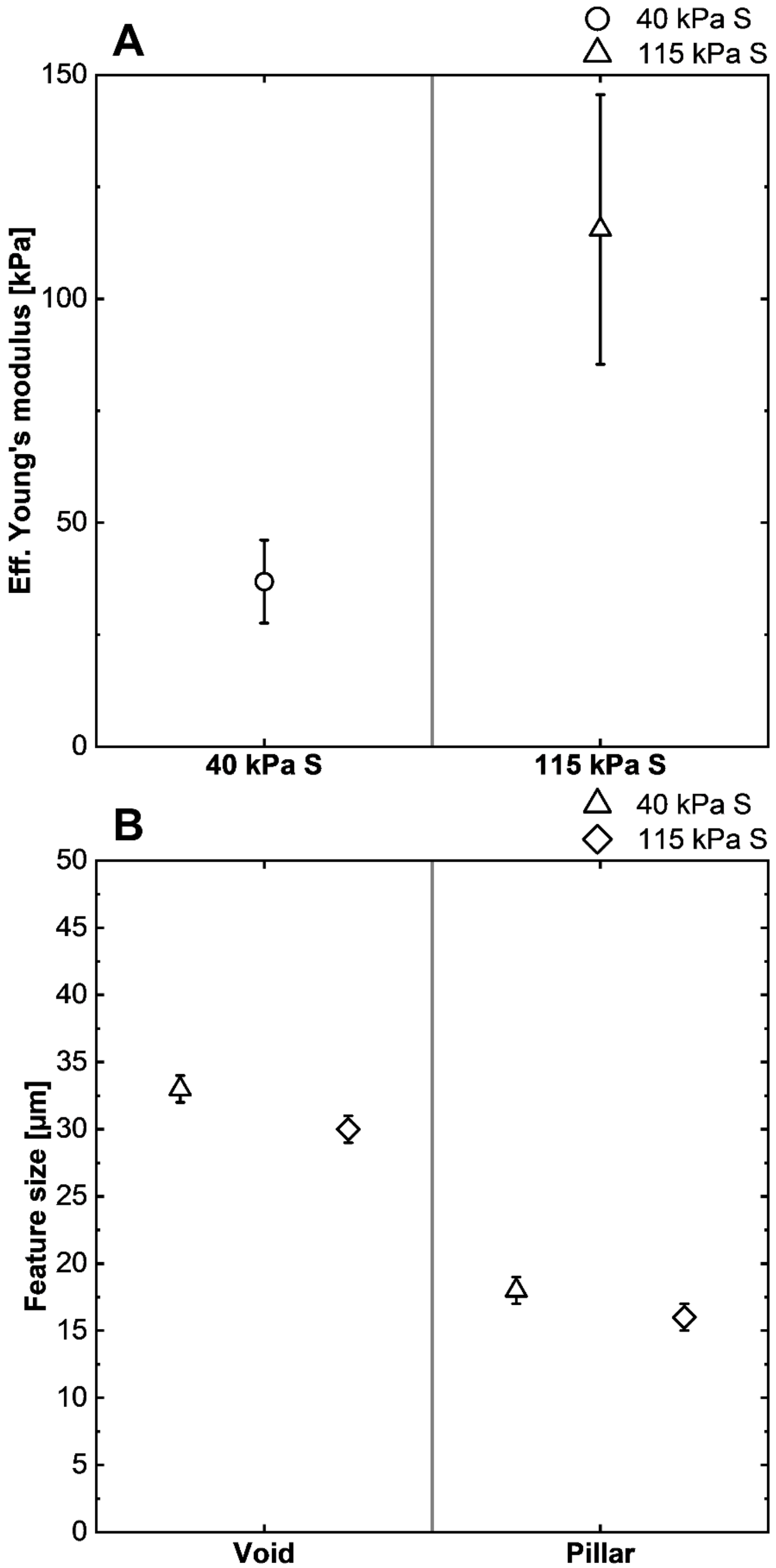

Figure S6: Parameters of the created hydrogel patches. *The effective Young's Moduli of the structured 15 wt.% (△), 20 wt.% (◇) hydrogel patches are depicted (**A**). The effective Young's Moduli were determined using a nano indenter (Optics11Life, Pavone) with a spherical tip as the cantilever probe (49.5 µm radius, 0.45 N/m stiffness). The feature sizes (**B**) either show the voids or pillars diameter of the structured 15 wt.% (△) and 20 wt.% (◇) hydrogel patches.*

PNT2							
**cytokine**	**time**	***Plastic***		***50 kPa***		***90 kPa***	
		concentrations in pg/ml					
		Mean	*SEM*	*Mean*	*SEM*	*Mean*	*SEM*
**IL-6**	**24h**	2851	89	2580	85	3281	116
	**48h**	6829	838	10084	82	7615	1401
	**72h**	4921	683	4346	664	4882	430
**Ang-1**	**24h**	53	0.1	41	1	52	n.a.
	**48h**	42	2	38	4	35	2
	**72h**	63	12	91	35	44	2
**Ang-2**	**24h**	1955	n.a.	595	92	1275	n.a.
	**48h**	55	27	76	38	96	11
	**72h**	2663	1560	2650	n.a.	n.a.	n.a.
**FGF**	**24h**	n.a.	n.a.	n.a.	n.a.	n.a.	n.a.
	**48h**	121	4	142	17	159	12
	**72h**	n.a.	n.a.	159	12	n.a.	n.a.
**CXCL8 (IL8)**	**24h**	587	63	561	18	623	42
	**48h**	1576	248	2035	238	1490	211
	**72h**	1037	279	752	193	766	24
**PECAM**	**24h**	n.a.	n.a.	n.a.	n.a.	n.a.	n.a.
	**48h**	209	15	286	35	210	26
	**72h**	n.a.	n.a.	n.a.	n.a.	n.a.	n.a.
**PIGF**	**24h**	n.a.	n.a.	n.a.	n.a.	n.a.	n.a.
	**48h**	12	1.1	18	2	14	2.2
	**72h**	n.a.	n.a.	n.a.	n.a.	n.a.	n.a.
**VEGF**	**24h**	1510	130	1914	110	1789	50
	**48h**	589	35	649	100	593	23
	**72h**	1393	166	1050	150	1280	50
**TNFα**	**24h**	n.a.	n.a.	n.a.	n.a.	n.a.	n.a.
	**48h**	5	0.3	6	1.5	6	0.8
	**72h**	n.a.	n.a.	n.a.	n.a.	n.a.	n.a.

Table S1: Cytokine expression of PNT-2 cells under conventional plastic and physio-elastic culture conditions. *Cytokines detected in conditioned media at 24, 48, and 72 h were quantified using the LEGENDplex™ Cytokine Array. The relevant cytokines and their secretion dynamics are presented as mean values derived from 12 measurements across three biological replicates, together with the corresponding standard error of the mean (SEM).*

MC3T3-E1							
cytokine	time	*Plastic*		*1 kPa*		*1600 kPa*	
		concentrations in pg/ml					
		Mean	*SEM*	*Mean*	*SEM*	*Mean*	*SEM*
**Ang-1**	**24h**	311	237	133	68	61	67
	**48h**	14	1	15	1	15	1.5
	**72h**	325	20	106	44	68	45
**Ang-2**	**24h**	7832	n.a.	13543	n.a.	1275	n.a.
	**48h**	55	27	76	38	96	11
	**72h**	4809	2233	8820	n.a.	n.a.	n.a.
**CXCL8 (IL8)**	**24h**	n.a.	n.a.	n.a.	n.a.	n.a.	n.a.
	**48h**	0.2	0.01	1.3	0.6	0.9	0.2
	**72h**	n.a.	n.a.	n.a.	n.a.	n.a.	n.a.
**PIGF**	**24h**	n.a.	n.a.	n.a.	n.a.	n.a.	n.a.
	**48h**	0.7	0.2	0.5	0.06	0.4	0.06
	**72h**	n.a.	n.a.	n.a.	n.a.	n.a.	n.a.

Table S2: Cytokine expression of MC3T3-E1 cells under conventional plastic and physio-elastic culture conditions. *Cytokines detected in conditioned media at 24, 48, and 72 h were quantified using the LEGENDplex™ Cytokine Array. The relevant cytokines and their secretion dynamics are presented as mean values derived from 12 measurements across three biological replicates, together with the corresponding standard error of the mean (SEM).*
